# Supervised learning of enhancer–promoter specificity based on genome-wide perturbation studies highlights areas for improvement in learning

**DOI:** 10.1093/bioinformatics/btae367

**Published:** 2024-06-13

**Authors:** Dylan Barth, Richard Van, Jonathan Cardwell, Mira V Han

**Affiliations:** School of Life Sciences, University of Nevada, Las Vegas, NV 89154, United States; Nevada Institute of Personalized Medicine, University of Nevada, Las Vegas, NV 89154, United States; School of Life Sciences, University of Nevada, Las Vegas, NV 89154, United States; Nevada Institute of Personalized Medicine, University of Nevada, Las Vegas, NV 89154, United States; Department of Medicine, University of Colorado School of Medicine, Denver, CO 80045, United States; School of Life Sciences, University of Nevada, Las Vegas, NV 89154, United States; Nevada Institute of Personalized Medicine, University of Nevada, Las Vegas, NV 89154, United States

## Abstract

**Motivation:**

Understanding the rules that govern enhancer-driven transcription remains a central unsolved problem in genomics. Now with multiple massively parallel enhancer perturbation assays published, there are enough data that we can utilize to learn to predict enhancer–promoter (EP) relationships in a data-driven manner.

**Results:**

We applied machine learning to one of the largest enhancer perturbation studies integrated with transcription factor (TF) and histone modification ChIP-seq. The results uncovered a discrepancy in the prediction of genome-wide data compared to data from targeted experiments. Relative strength of contact was important for prediction, confirming the basic principle of EP regulation. Novel features such as the density of the enhancers/promoters in the genomic region was found to be important, highlighting our lack of understanding on how other elements in the region contribute to the regulation. Several TF peaks were identified that improved the prediction by identifying the negatives and reducing False Positives. In summary, integrating genomic assays with enhancer perturbation studies increased the accuracy of the model, and provided novel insights into the understanding of enhancer-driven transcription.

**Availability and implementation:**

The trained models, data, and the source code are available at http://doi.org/10.5281/zenodo.11290386 and https://github.com/HanLabUNLV/sleps.

## 1 Introduction

Enhancers are regulatory elements that affect gene expression through the direct physical interaction with promoters. Various context-specific transcription factors (TFs) gather at the enhancer and the promoter where the general TFs bind to activate transcription. This general model has been validated through many studies (Tolhuis *et al.* 2002, Palstra *et al.* 2003), but challenged in certain contexts (Alexander *et al.* 2019). A central question surrounding the interaction between regulatory elements and their target genes is what principles determine which elements regulate any given gene and how such specificity is achieved. One working model is the Activity-by-Contact (ABC) model (Fulco *et al.* 2019). The ABC model uses H3K27ac, DNase I hypersensitive sites (DHS), and Hi-C to rank the importance of the enhancer to the regulation of a gene as proportional to its activity multiplied by contact. Fulco *et al.* (2019) performed CRISPRi flowFISH experiments and showed the utility of the model by predicting functional enhancer–promoter (EP) pairs with state-of-the-art accuracy. While the ABC model is elegant, and works better than any alternative approach, several important biological factors are ignored in the model, such as the combination of TFs or chromatin marks other than H3K27ac. It also only relies on direct contacts between EPs and ignores other interactions in the region. Since Fulco *et al.* (2019) there have been several large scale studies that deployed CRISPRi to perturb multiple enhancers and measured the effects on gene expression often through single-cell RNA-seq (Gasperini *et al.* 2019, Schraivogel *et al.* 2020). The accumulation of such data based on large enhancer perturbation studies provides an opportunity to train a supervised model on verified enhancer data and gain a more detailed understanding of EP specificity.

Here, we utilized the largest CRISPRi experiment to date (Gasperini *et al.* 2019) to predict functional EP pairs that showed significant down-regulation after perturbation. Note that this aim is different from studies that predicted enhancer elements without relations to its target (Erwin *et al.* 2014, Yang *et al.* 2017). It is also different from studies that came before high-throughput perturbation experiments such as TargetFinder (Whalen *et al.* 2016) or JEME (Cao *et al.* 2017) that predicted chromatin interactions (chromatin loops) without distinguishing functional effects on gene expression. The most relevant studies would be Schraivogel *et al.* that built a supervised model with less number of features (Schraivogel *et al.* 2020), or Bergman *et al.* (2022) and Martinez-Ara *et al.* (2022) that systematically examined the compatibility of enhancers and promoters in human and mice, respectively. However, the latter two studies utilized data from episomal high-throughput reporter assays that are different from CRISPRi experiments that occur in native genomic context. By training a machine learning algorithm on the CRISPRi experiments of Gasperini *et al.* (2019), we could integrate genomic assays measured on the candidate enhancer and target promoter in native genomic context into the feature space, such as the 3D chromatin contact and various TF ChIP-seq. Based on this framework, we aimed to predict functional EP relationships and learn the important features that contribute to EP specificity, especially in cases of direct and indirect contact.

## 2 Materials and methods

### 2.1 Candidate EP pairs and feature engineering

To identify the potential candidate EP pairs, we utilized the ABC software (Fulco *et al.* 2019) that identified all enhancer and target promoter pairs within a 5-Mb window for every gene in the genome. More details on candidate EP generation are described in the Supplementary Methods. We then overlapped the candidate EPs with experimental data from the enhancer perturbation study by Gasperini *et al.* (2019). The feature matrix has rows representing EP pairs and columns representing features. The features included measures of histone modification (H3K27ac, H3K4me3, H3K27me3) around the enhancer, same set of histone modifications around the TSS, expression level of the target gene, genomic distance between the enhancer and the TSS, KR-normalized Hi-C contact measure between the enhancer and the TSS, relative strength of the chromatin contact compared to other enhancers or other TSS nearby, chromatin density of the region represented by the total number of enhancers and TSS in the neighborhood, 250 TF presence at the enhancer and at the TSS based on ChIP-seq from ENCODE. The final matrix contained 542 features for 65 374 EP pairs. We also used non-negative matrix factorization (NMF) to generate clustered profiles of correlated TF binding on enhancers and TSS. The full list of features can be found in Supplementary Table S1. More details on generating each feature are described in the Supplementary Methods.

### 2.2 Training data, validation data, and independent test data

To compile experimentally verified positive and negative EP pairs that we used for training, validation, and test, in addition to Gasperini2019 (Gasperini *et al.* 2019), we also overlapped the candidate EPs with experimental data from independent enhancer perturbation studies, Fulco2019 (Fulco *et al.* 2019) and Schraivogel2020 (Schraivogel *et al.* 2020). The output we predicted is a binary class label that was assigned based on the experimental results that is positive only if blocking said enhancer using a CRISPRi gRNA resulted in significant down-regulation of the gene (adjusted *P*-value < .1). Significant up-regulation of the genes was not considered as positive.

The compiled dataset from Gasperini2019 was divided into training and test, by setting aside chromosomes 5, 10, 15, and 20 as test sets. The EP pairs that have “NA” in the column “pValueAdjusted” were filtered out, to result in a total of 29 291 EP pairs for training, and 4274 EP pairs for test. Independent test datasets were generated by compiling the data from Fulco2019, Schraivogel2020 and the associated features in the same manner. For Fulco2019, we excluded distal promoter-gene pairs as described in the article, leading to 104 positive EPs out of 3857. For Schraivogel2020, we used the same filtering criteria described in the article, leading to 64 positives out of 918. The counts are slightly smaller than those in the original studies due to rows that were excluded because of missing overlap or missing feature values. The remaining training data of Gasperini2019 (chromosomes other than 5, 10, 15, 20) were then divided into a 4 × 4 nested blocked cross-validation (CV) scheme (using *StratifiedGroupKFold*), where blocking was based on the groups defined by at least 5 Mb of gap separating any of the enhancers or TSS in the data. Any enhancer–TSS pairs that are within the boundaries were assigned to the same group, and all data within the same group were assigned into a single fold, ensuring separation by genomic distance, and thus independence between data across each fold (Xi and Beer 2018, Whalen *et al.* 2022). The grouping of the data and folds can be found in Supplementary Table S2. The outer folds were used for evaluation of performance, while the inner folds were used for hyper-parameter optimization.

### 2.3 Machine learning pipeline

We used extreme gradient boosting (XGB) for learning (Chen and Guestrin 2016). We explored other algorithms that did not give us similar or better performance, so we do not report those results. Scaling, dimension reduction, feature selection, and hyper-parameter optimization were done within the training split of the outer fold. Test split of the outer fold was used for unseen performance evaluation for the pipeline trained on the training split. We did not retrain the final pipeline on combined folds. We opted to evaluate the four different pipelines produced by 4-folds separately, to get a measure of uncertainty around the performance evaluation.

Within each training split, we scaled the features to be between 0 and 1. We did feature selection using Boruta with SHAP (SHapley Additive exPlanations) values on XGB trees trained through a preliminary optimization (Kursa and Rudnicki 2010). The hyper-parameters were optimized on the inner fold CVs using Optuna (Akiba *et al.* 2019), with pruning and early stopping based on the validation set metric. Due to the extreme imbalance in the data (601 positive out of 33 565 total), we used mean average precision (MAP) averaged across the inner folds as the metric to maximize during CV. The search space we explored and the final optimized parameters can be found in Supplementary Table S3. The SHAP values were calculated using the tree SHAP implementation in XGB. SHAP values for the four test folds were combined through concatenation.

### 2.4 EP relations in the Chromatin Interaction Network

We generated a Chromatin Interaction Network (ChIN) based on Hi-C contact maps centered around the promoter for each gene, where each node was an enhancer or promoter, and the edge weights were equal to the KR-normalized Hi-C contact between those nodes. We simplified the network by only leaving edges with significant chromatin loops using FitHiC2 (Kaul *et al.* 2020). The network was then further filtered by only keeping nodes of enhancers that were tested in Gasperini2019 (Gasperini *et al.* 2019) (Supplementary Fig. S1). From these final ChINs, the relationship between each EP pair was determined following Song *et al.* (2019). EPs that were directly connected with a significant Hi-C loop (distance 1) were labeled *e*_1_, EPs that were connected through an *e*_1_ enhancer node (distance 2) were labeled *e*_2_, EPs that were connected through two enhancer nodes (distance 3) were labeled *e*_3_. EPs within the same Hi-C window were labeled *e*_0_, and EPs that were more than three edges apart (distance >3) or enhancers not part of the ChIN of the promoter were labeled *e_inf_*.

## 3 Results

### 3.1 Integration of additional genomic features improves prediction of functional EP pairs by learning the genes insensitive to enhancer perturbations

By integrating the TF ChIP-seq from ENCODE, with the experimental study by Gasperini *et al.*, we could train a supervised XGB model that predicts functional EP pairs. The overall MAP from the inner fold validations were around 0.27–0.31, and the outer fold average precision on the test folds ranged from 0.22 to 0.32 (Table 1a). Although the outer test fold performance has a larger variance, the overall performance between the CV and the test is comparable, showing that we are not overfitting to the training data. We also had an independent test set from Gasperini2019 consisting of chromosomes 5, 10, 15, 20 that were set aside from the nested CV design, and the test performance on those data also showed comparable values of average precision between 0.28 and 0.37, again confirming no overfitting (Table 1c).

**Table 1.** Performance of the XGB model: performance based on (a) inner fold CV, (b) outer fold test data for each of the four models trained on the 4-folds. (c) Test data from Gasperini *et al.* (2019). Chromosomes 5, 10, 15, 20. (d) Test data from Fulco *et al.* (2019). (e) Test data from Schraivogel *et al.* (2020).

(a)	Inner folds CV		
	Model	Data	Aucpr
	model1	fold1 trainsplit	0.299
	model2	fold2 trainsplit	0.271
	model3	fold3 trainsplit	0.312
	model4	fold4 trainsplit	0.293

(b)	Outer folds test					
	Model	Data	Aucpr	Avg precision	Balanced accuracy	*f*1 score
	model1	fold1 testsplit	0.217	0.222	0.763	0.125
	model2	fold2 testsplit	0.319	0.321	0.827	0.329
	model3	fold3 testsplit	0.291	0.294	0.785	0.156
	model4	fold4 testsplit	0.299	0.303	0.822	0.293

(c)	Gasperini2019 Test (chr 5, 10, 15, 20)					
	Model	Data	Aucpr	Avg precision	Balanced accuracy	*f*1 score
	model1	Gasperini Test	0.274	0.284	0.823	0.289
	model2	Gasperini Test	0.323	0.330	0.825	0.306
	model3	Gasperini Test	0.370	0.374	0.827	0.315
	model4	Gasperini Test	0.312	0.316	0.823	0.293

(d)	Fulco2019					
	Model	Data	Aucpr	Avg precision	Balanced accuracy	*f*1 score
	model1	Fulco	0.692	0.693	0.844	0.629
	model2	Fulco	0.671	0.672	0.836	0.498
	model3	Fulco	0.670	0.671	0.839	0.549
	model4	Fulco	0.670	0.671	0.838	0.537

(e)	Schraivogel2020					
	Model	Data	Aucpr	Avg precision	Balanced accuracy	*f*1 score
	model1	Schraivogel	0.462	0.474	0.809	0.503
	model2	Schraivogel	0.470	0.476	0.795	0.446
	model3	Schraivogel	0.466	0.471	0.800	0.464
	model4	Schraivogel	0.461	0.466	0.803	0.476

**Figure 1.**
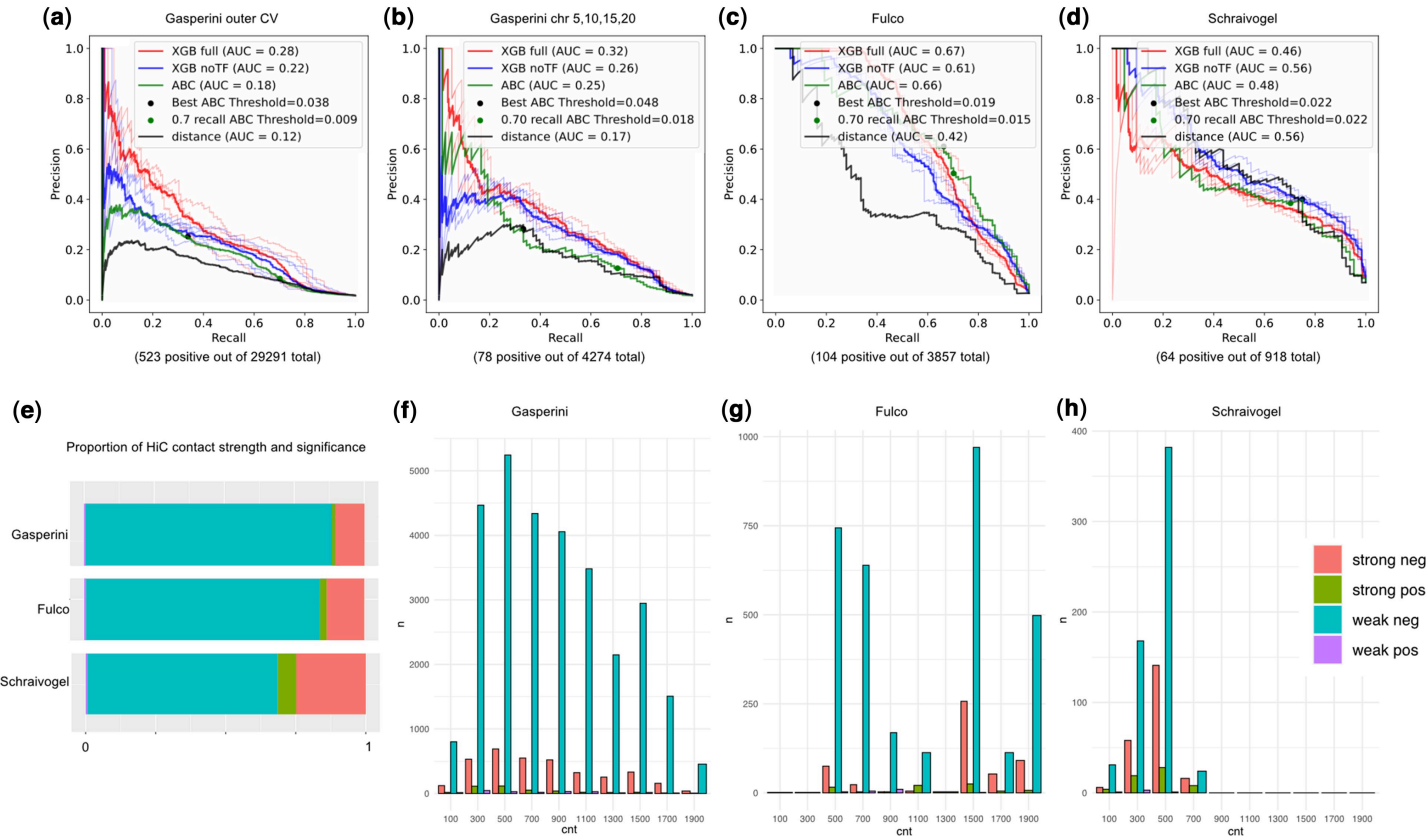
Comparison of model performance and data distribution. Precision–recall curves of various models on data from (a) Gasperini2019 outer test folds, (b) Gasperini2019 chromosomes 5, 10, 15, 20. (c) Fulco2019 and (d) Schraivogel2020. The translucent lines are the performance of each model trained on 4-folds. The solid line is the performance combined across the folds. In all cases, except for Schraivogel2020, the XGB full model trained on the integrated genomic data outperforms the XGB simple model without TF features, ABC model and distance-based model. (e) Comparison of Hi-C contact strength and significance. EP pairs are divided into strong contact (≥0.005) and weak contact (<0.005) and positive EPs (significant down-regulation) and negative EPs (not significant). (f–h) EPs are binned by the total count of elements, both enhancers and TSS, in the neighborhood. Legends weak neg: negative with weak contact, strong pos: positive with strong contact. Note that targeted experiments have higher proportion of both strong neg and strong pos, indicating stronger contact compared to the genome-wide data

Integrating various genomic measures as features in the learning increased the performance of functional EP prediction compared to the ABC model or distance-based prediction. On the Gasperini2019 dataset, we achieved an average precision of around 0.32. Considering the challenge of the dataset, which is an extremely imbalanced dataset with <2% positive rate (78 positive out of 4274 total in the chr 5, 10, 15, 20 test data), we see a meaningful increase in the performance (Fig. 1a and b). The effect of TF ChIP-seq quality on the performance is reported in Supplementary Fig. S2. With the Fulco2019 dataset, which has a higher positive rate of about 2.7% (104 positive out of 3857 total), the performance reaches 0.67 average precision (Table 1d). We saw a more modest increase in the performance compared to ABC (Fig. 1c). With the Schraivogel2020 dataset, we actually see a lower performance compared to ABC or distance, that we described in more detail in Section 3.2.

We compared the experimentally validated EP pairs of Gasperini2019 with the predictions from XGB at recall = 0.7. There were 16 positive EPs and six null (negative) enhancers that were validated. Of the 16 positive EPs, XGB predicted 15 as positive and one as negative. For the six null enhancers, there were 95 EP pairs across multiple genes that were measured in the target region. Out of the 95 EP pairs, 87 were predicted as negative and eight were predicted positive, leading to a False Positive rate of 8/(8 + 87) = 0.084 (Supplementary Table S4).

We also compared the EP pairs exclusively captured by XGB versus ABC and examined if they were enriched in terms of regulatory features. The exclusively True Positive counts were the same between XGB and ABC (35 versus 35), but the exclusively True Negative counts were larger in the XGB than the ABC (2395 versus 974; Supplementary Table S5). In general, the EPs exclusively correctly identified by XGB were not characteristic positives or negatives (Supplementary Fig. S3).

Overall, there was a discrepancy in the performance on the unbiased data of Gasperini2019 versus the targeted data of Fulco2019 and Schraivogel2020. As can be seen in Fig. 1e–h, the distribution of positive cases and the Hi-C contact strengths are different between the genome-wide data and the targeted data. The targeted experimental design is focused on systems that will likely yield positive results, as it should be, and thus the individual regions selected have higher target gene expression, stronger Hi-C contact with candidate enhancers, and higher positive rates (Fig. 1e–h, Supplementary Fig. S4). In fact, the majority of the targeted genes have at least one positive enhancer in Fulco2019 and Schraivogel2020. On the other hand, with the Gasperini2019 dataset, the enhancers were selected based on their features, but the target genes were assayed using single-cell RNA-seq, thus there are overwhelmingly larger number of negative genes and weak contact. We found that the XGB model trained on this dataset is achieving better performance by learning to reduce the false positive rate by distinguishing the negative genes that are insensitive to enhancer regulation. Looking at the confusion matrix (see Table 2), we can confirm that the increase in performance in the Gasperini data is due to the reduction in the false positives in XGB compared to ABC at the same recall. The TF binding at the promoters are important in achieving this discrimination, and will be discussed in Section 3.4. The individual decision trees from the trained XGB model can be found in Supplementary Table S6.

**Table 2.** Confusion matrices: confusion matrices at 0.70 recall for (a) XGB and (b) ABC model applied to each dataset.

(a)	XGB (recall = 0.7)			(b)	ABC (recall = 0.7)		
	Gasperini CV				Gasperini CV		
		pred neg	pred pos			pred neg	pred pos
			
	neg	25 698	2957		neg	24 707	3948
	pos	156	367		pos	156	367
	Gasperini Test (chr 5, 10, 15, 20)				Gasperini Test (chr 5, 10, 15, 20)		
		pred neg	pred pos			pred neg	pred pos
			
	neg	3959	216		neg	3795	380
	pos	23	55		pos	23	55
	Fulco				Fulco		
		pred neg	pred pos			pred neg	pred pos
			
	neg	3698	55		neg	3682	71
	pos	31	73		pos	31	73
	Schraivogel				Schraivogel		
		pred neg	pred pos			pred neg	pred pos
			
	neg	756	70		neg	757	69
	pos	19	45		pos	19	45

### 3.2 Integration of TF binding does not improve prediction when conditioned on the presence of at least one positive enhancer regulating the target promoter

**Figure 2.**
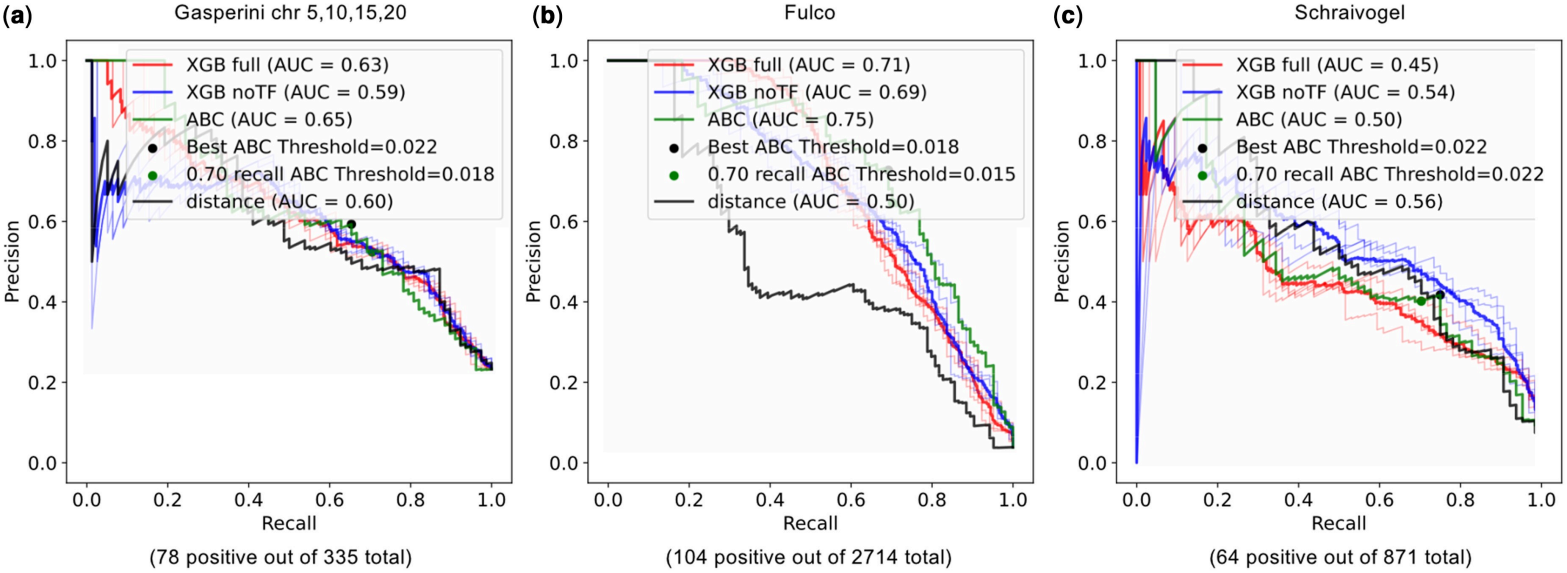
Comparison of model performance for genes with at least one positive enhancer. Precision–recall curves of various models on data filtered for genes with at least one positive enhancer from (a) Gasperini2019 chromosomes 5, 10, 15, 20, (b) Fulco2019 and (c) Schraivogel2020. In all cases, the XGB full model trained on the integrated genomic data does not outperform the XGB simple model without TF features nor the ABC predictions

Based on the observation that the integrated model performed better on the genome-wide data, but not as well on targeted data, we wanted to know whether the additional features would be helpful in the prediction on Gasperini2019 data when filtered in a similar way. We compared the full model versus the reduced model without TF features on the whole data and the data filtered to have genes with at least one significant enhancer. As described in Section 3.1, on the whole data, adding TF features consistently increased the AUC by about 0.06 for both Gasperini2019 and Fulco2019 (Fig. 1a–c). Note here that the Schraivogel2020 is already filtered the same way based on the original paper, so we do not see the same increase in performance (Fig. 1d). In contrast, when conditioned on the presence of at least one positive enhancer for the gene, the additional TF binding information in the full model did not increase the performance and sometimes even slightly decreased it compared to the reduced model with no TF information (Fig. 2a–c). Once we know that there is a large effect enhancer that regulates the gene, chromatin and contact is the only information that is necessary to find the enhancer, and TF peaks, at least the 250 ones that we included, did not improve the prediction.

### 3.3 Feature importance of the learned model corroborates the ABC model and importance of relative contact strength

**Figure 3.**
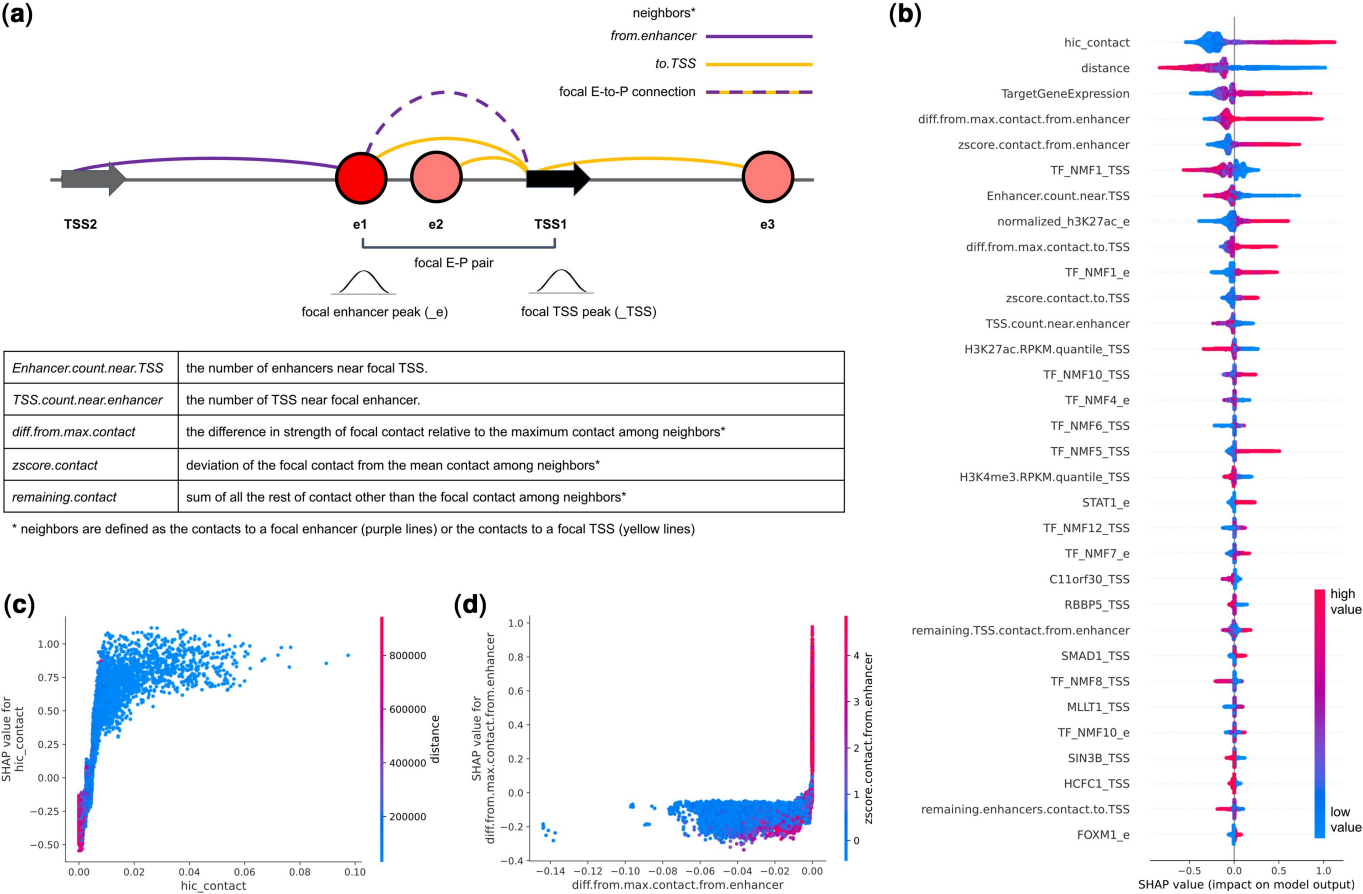
Feature importance. (a) Contact features are defined for the focal contact relative to the neighboring contacts. Neighbors are either all the contacts originating from the enhancer, or all the contacts reaching the TSS. ChIP-seq features are defined based on peaks at the enhancer (_e) or peaks at the TSS (_TSS). (b) Top 32 most important features ranked by their mean absolute SHAP values. Samples appear as points along the horizontal axis and colors correspond to the value of that feature. The *x*-axis predicts the outcome as positive or negative. Note high values of Hi-C contact predict positive outcome (right of the horizontal axis), while low values of distance predict positive outcome (right of the horizontal axis). (c) Hi-C contact shows positive trend with SHAP values, and negative correlation with distance. (d) If the focal contact is the strongest among all contacts originating from the enhancer (i.e. if the difference to the max is zero), then the EP is predicted to be positive

To understand where the increased performance for the genome-wide data is coming from, we calculated the SHAP values (Lundberg and Lee 2017). Positive SHAP value means the feature leads the model to predict positive (functional) outcome. Negative SHAP value means the feature leads the model to predict negative (nonfunctional) outcome. Note that positive EP pairs will tend to have large negative effect size in their differential gene expression after perturbation. To summarize the SHAP values that we obtained, stronger Hi-C contact, shorter distance between enhancer and target TSS, various measures of relative contact rank in the surrounding genomic region, higher target gene expression, higher H3K27ac peak at the enhancer, and less number of enhancers and promoters nearby, all contributed to the model to predict positive EP pairs (Fig. 3b). A larger figure listing the top 100 features can be found in Supplementary Fig. S5. Some of the features (e.g. *TargetGeneExpression*) were related to the power to detect, and we could see higher performance once we filtered out lowly expressed genes (Supplementary Table S7). The factors that are components for the ABC model such as the Hi-C contact (Fig. 3c, Supplementary Fig. S6), and the H3K27ac chromatin marks at the enhancer were at the top of the feature ranking, confirming the validity and power of the ABC model. In addition, we confirmed that the enhancer-centric contact rank is also important in addition to the TSS-centric contact rank (Fig. 3a), as has been reported in the adapted ABC model by Hecker *et al.* (2023). Whether the TSS is the TSS with the maximum contact among all the TSS that are around the focal enhancer (*diff.from.max.contact.from.enhancer*) was as important or even more important than whether the enhancer was in maximum contact among the all the enhancers around the TSS (Fig. 3d).

### 3.4 Strong H3K27ac, EMSY, and HCFC1 present at the TSS predict genes that are insensitive to enhancers

The SHAP analysis highlighted *NMF1_TSS*, H3K27ac at the TSS, C11orf30 (EMSY) and HCFC1 (HCF1) at the TSS as contributing to negative prediction, meaning that the model is less likely to predict a functional EP pair for that TSS (Fig. 3b). Here, note that the H3K27ac mark at the TSS (*H3K27ac.RPKM.quantile_TSS*) shows an opposite contribution compared to the H3K27ac mark at the enhancer (*normalized_H3K27ac_e*). The presence of a strong activating chromatin mark at the TSS being associated with less enhancer effect (Supplementary Fig. S7) is consistent with several reports on strong promoters. Bergman *et al.* showed that promoters of ubiquitously expressed genes have higher H3K27ac marks at the promoter and are less responsive to distal enhancers (Bergman *et al.* 2022). Similarly, in both Gasperini2019 and Fulco2019, the experiments showed that housekeeping genes are depleted from their positive EP pairs (Fulco *et al.* 2019, Gasperini *et al.* 2019), indicating that housekeeping genes are less influenced by enhancers, and their transcription is sufficiently driven by promoters alone.

**Figure 4.**
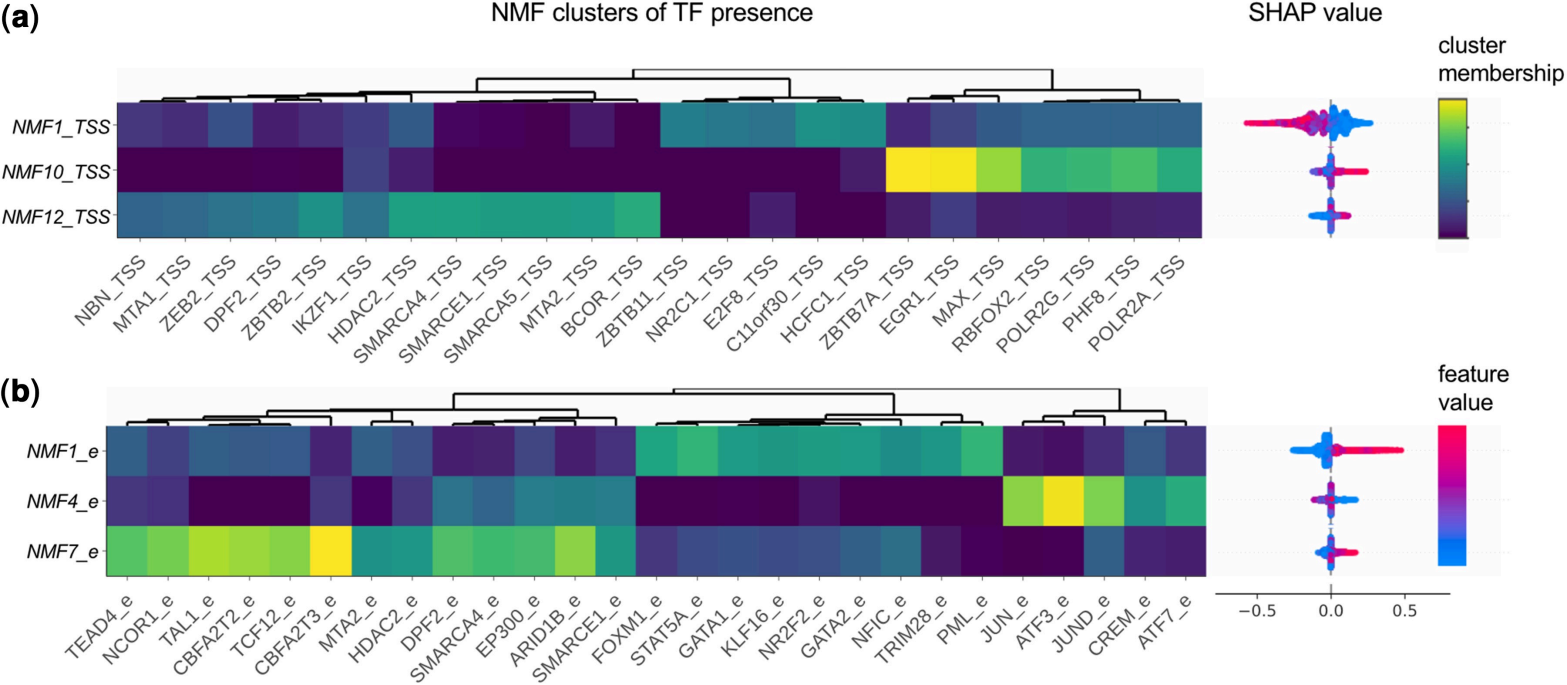
Clusters of correlated TF presence. Clusters (*k* = 12) of TF co-binding were identified using non-negative matrix factorization (NMF) applied to TF presence matrix at the (a) TSSs and (b) enhancers separately. The TF membership (*H* matrix) for top three clusters important for prediction is shown, next to their SHAP values

The TF presence at the TSS contributed to further improve the prediction of insensitive genes. The most important TF feature was the *NMF1_TSS* cluster at the TSS, that predicted negative EP pairs. *NMF1_TSS* includes a broad array of TFs co-binding at the TSS, including HCFC1, C11orf30, E2F8, NR2C1, and ZBTB11 (Fig. 4a). The full NMF membership matrix describing the TF co-binding clusters for TSS and enhancers can be found in Supplementary Fig. S8. C11orf30 (EMSY) and HCFC1 also show up as important individual TFs at the TSS, both contributing to nonfunctional EP prediction (Fig. 3b, Supplementary Fig. S9). EMSY and HCFC1 are known to have both repressing and activating functions (Hughes-Davies *et al.* 2003, Wysocka *et al.* 2003), but in this data, both EMSY and HCFC1 peak at the TSS were associated with higher H3K27ac marks and H3K4me3 marks at the TSS, and more active promoters (Supplementary Fig. S10b–e). This was also confirmed through the hierarchical clustering of SHAP values across features. The clustering shows that H3K27ac, H3K4me3, and HCFC1 at the TSS are correlated in their effect on prediction (Supplementary Fig. S10a). Note that we do not consider up-regulation as significant, so repressive function would not be learned from the data. Correlated features of H3K27ac, H3K4me3, HCFC1, and EMSY peaks at the TSS together indicate potent self-sufficient promoters and lead to decreased prediction of functional enhancers affecting the target gene.

### 3.5 TFs present at the enhancers and promoters predict functional EP pairs

On the other hand, numerous TFs found at the enhancer regions were predictive of the functional EP pairs in the positive direction. The most important TF feature at the enhancer was *NMF1_e* that is a TF cluster that includes presence of KLF16, GATA1, GATA2, NR2F2, STAT5A, NFIC, PML, FOXM1 at the enhancer (Fig. 4b). TFs found to be highly important when learning on individual TF peaks without the clustered NMF peaks also included the well-known hematopoietic and/or cancer-related TFs: STAT1, STAT2, STAT5A, GATA2, FOXM1, etc. (Supplementary Figs S11 and S12) (Crispino and Horwitz 2017, Fasouli and Katsantoni 2021).

In addition to these TFs, there were several TF clusters that predicted positive EPs. *NMF10_TSS* at the promoter included RNA Pol II, RBFOX2, and predicted positive EPs. The *NMF12_TSS* at the promoter and the *NMF7_e* at the enhancer were enriched in several common proteins that are part of the NuRD and SWI/SNF chromatin remodeling complexes, and both clusters predicted positive EPs.

Most TF features at the enhancers were positively predictive of functional EP pairs. One of the few TF features at the enhancers that contributed to negative prediction was the *NMF4_e* cluster at the enhancer that included proteins that are members of the AP-1 complex. Given the role of AP-1 complex as an activator in gene regulation, we hypothesize that the negative prediction here has to do with redundancy of the enhancers bound by AP-1. Similar to how negative prediction in the TSS had to do with strong promoters that are insensitive to enhancer regulation, the enhancers bound by AP-1 may be more robust enhancers that are part of a hub of redundant EP interactions (Phanstiel *et al.* 2017, Seo *et al.* 2021). Perturbation of such enhancers is likely to have less effect on the gene expression because there will be other enhancers that are part of the hub that can provide buffering of the perturbed effect.

### 3.6 The density of the enhancers and promoters within the chromatin landscape is a factor modulating the detection of functional EPs

**Figure 5.**
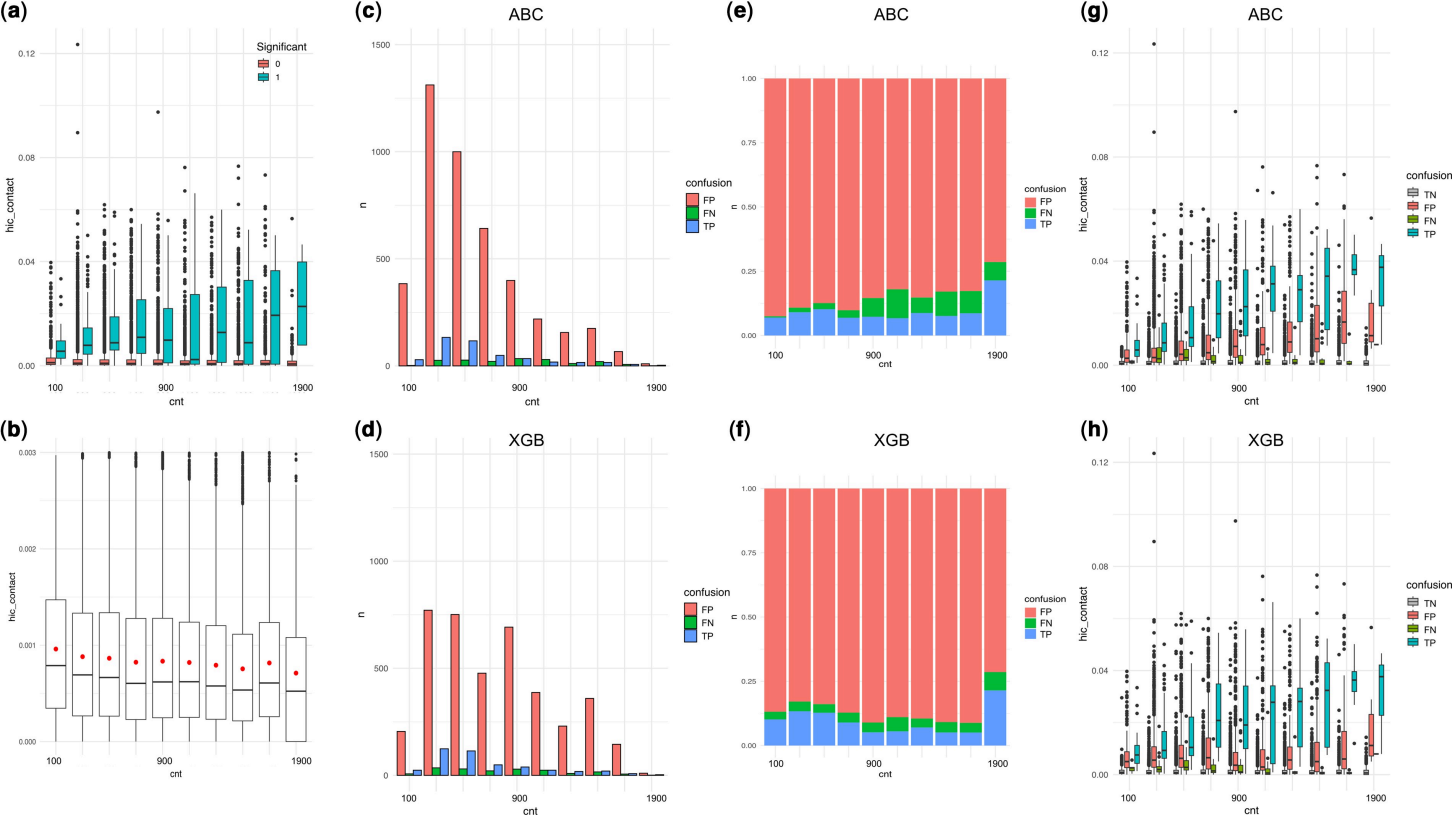
EP detection and regulatory element density in the region. (a) Significant EPs called by Gasperini2019 show stronger Hi-C contact in high-density regions. (b) Mean and median Hi-C contact is lower in high-density regions. (c, d) Absolute counts of False Positives (FP) and False Negatives (FN) and True Positives (TP) for ABC and XGB. (e) Proportion of False Negatives increase while False Positives decrease with density in ABC models. (f) Proportion of False Positives and False Negatives both increase with density in XGB models. (g, h) Hi-C contact strength for True Negatives (TN), FP, FN, and TP in (g) ABC and (h) XGB models

The total number of enhancers near the focal TSS (*Enhancer.count.near.TSS*) and the total number of TSS near the focal enhancer (*TSS.count.near.enhancer*) were both important features that were negatively associated with functional EP pairs (Fig. 3b). It is unlikely that there are less functional interactions in a region where there are many more open chromatin and potential interacting partners of enhancers and TSS. Instead, we interpret this as highlighting the difficulty of detection around a region that is dense in regulatory information. This loss of power occurs at two levels. At the experimental level, there is loss of power in calling positive effects as evidenced by the pattern of stronger Hi-C value required for positive pairs in regions of high density (Fig. 5a). At the algorithmic level, there is difficulty in predicting positive EP pairs as evidenced by the pattern of more False Negatives observed in regions of high density (Fig. 5c–h). If one looks at the Hi-C contact strength, the contacts are actually weaker in dense regions (Fig. 5b). It is possible that this is an artifact of having a larger denominator that pulls the median and mean of the contact strengths lower. Despite this lower average Hi-C, if one looks at the positive calls made by Gasperini *et al.* (2019), the EPs need to have a stronger Hi-C contact to be called positive in high-density regions (Fig. 5a). One potential explanation is that there is higher chance of redundancy in dense regions, such that only perturbations of a stronger contact will have an observable effect. In prediction, we see higher False Negatives in dense regions in both ABC and XGB predictions, but the missed cases in dense regions are worse in ABC models (Fig. 5e and f). This is likely due to the fact that as density increases, the denominator of the ABC grows larger and the overall ABC score grows smaller, while the threshold is fixed, leading to more negative calls.

### 3.7 Sorting the enhancers based on contact reveals a class of atypical enhancers that reside in high-density genomic regions

Machine learning revealed that Hi-C contact between the EP is the most important feature in predicting functional EP pairs. Nevertheless, there are a non-negligible number of positive functional pairs that are not in strong contact. Gasperini *et al.* discussed this observation as underscoring the difficulty of the prediction task (Gasperini *et al.* 2019). To understand the difference between functional EPs that are in strong contact, and functional EPs that are not in contact, we split the data into two classes based on the distance in the ChIN. We call the direct contact EPs e1minus (encompassing *e*_1_ and *e*_0_), and we call the indirect EPs that are not in direct contact e2plus (*e*_2_, *e*_3_, *e_inf_*). We trained the same machine learning pipeline on these two datasets. Overall, limiting the data to e1minus EPs with direct contacts (348/6304 = 5.5% positive rate) made the prediction easier, reaching average precision that is about 30% higher than what we saw with the whole data (Supplementary Table S8a). In contrast, the training and prediction of e2plus EPs without direct contact (175/22 874 = 0.4% positive rate) were extremely challenging, resulting in overfitting based on the difference seen between CV MAP and Test MAP, and an order of magnitude lower average precision (Supplementary Table S8b).

**Figure 6.**
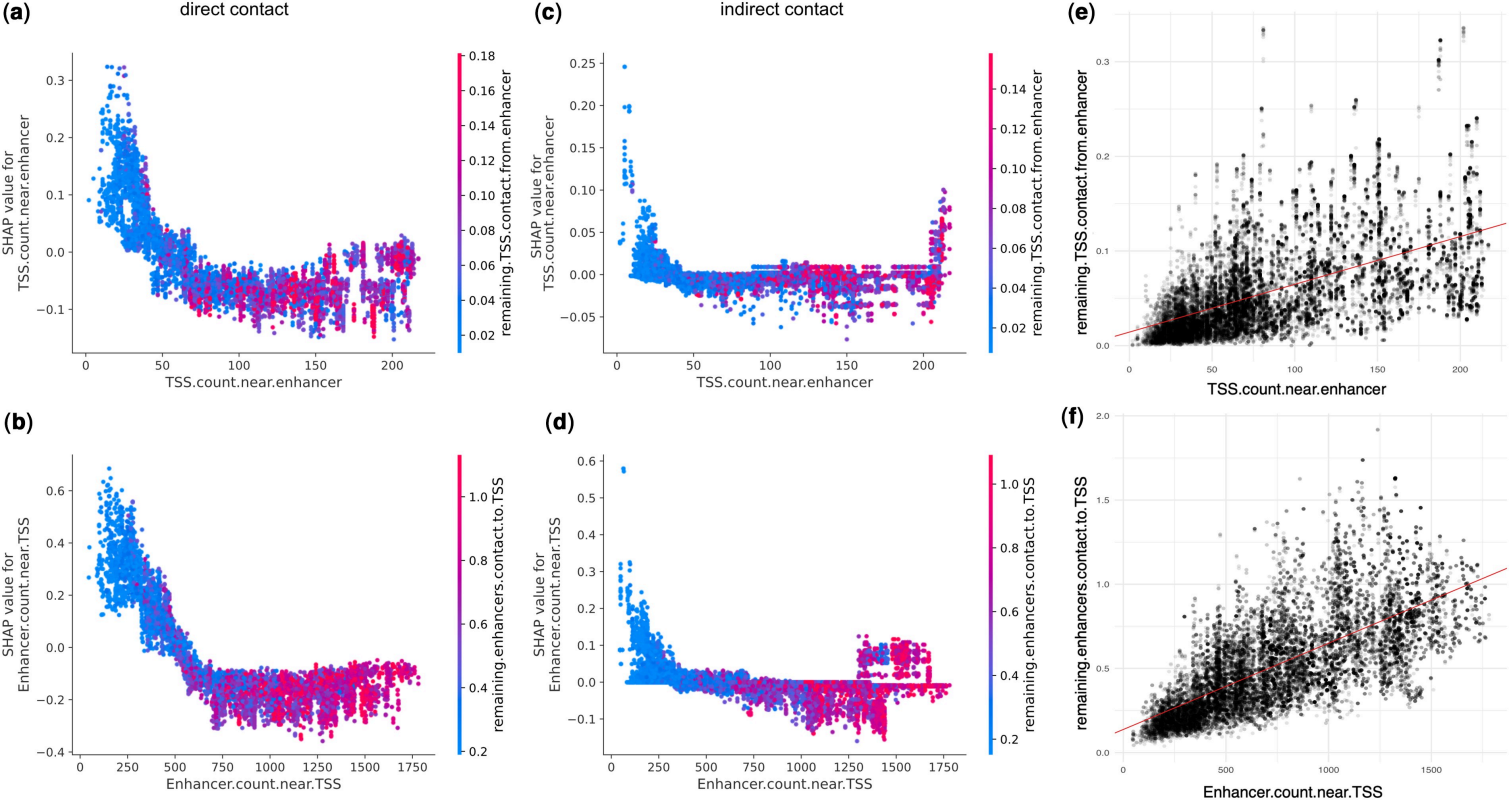
Indirect contacts and regulatory element density. (a, b) For EPs with direct contacts, both *enhancer.count.near.TSS* and *TSS.count.near.enhancer* showed negative trend with SHAP values, indicating they are less likely to be predicted positive when there are many other enhancers or TSS nearby. (c, d) For EPs with indirect contacts, there is a subset of EPs that are more likely to be predicted positive when there are many other enhancers or TSS nearby. (e, f) *remaining*. *TSS.contact.from.enhancer* and *remaining.enhancers.contact.to.TSS* summarize alternative contacts in the neighborhood, and are positively correlated with the density of enhancers and TSSs in the genomic region

Despite the difficulty in prediction, the SHAP values of top features were largely similar (Supplementary Fig. S13). The exceptions were the features representing the strength of other remaining contacts surrounding the EP (Fig. 3a). *remaining*. *TSS.contact.from.enhancer* is the sum of Hi-C contacts between all the other TSS and the focal enhancer and *remaining.enhancers.contact.to.TSS* is the sum of Hi-C contacts between all the other enhancers and the focal TSS. Both features are also correlated with the EP density of the region (Fig. 6e and f). Only in the indirect e2plus EPs, we saw a subset of EP pairs that showed opposite trend from the rest of the data, i.e. stronger positive prediction when the *remaining.contact* is larger (Fig. 6c and d). The pattern is replicated in the whole data (Supplementary Fig. S14), but not in the direct contacts.

**Figure 7.**
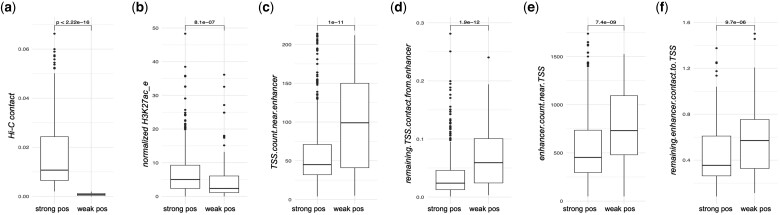
Features that are different between positive EPs with strong contact versus weak contact. strong pos, positive EP with strong contact (Hi-C ≥ 0.002); weak pos, positive EP with weak contact (Hi-C < 0.002) (a) Hi-C contact. (b) normalized H3K27ac at the enhancer. (c) TSS count near the enhancer. (d) remaining TSS contact from the enhancer. (e) enhancer count near the TSS. (f) remaining enhancer contact to the TSS.

Based on this analysis, we went back to the original data from Gasperini2019, to check if the same features show difference between the positive cases that have weak contact (Hi-C < 0.002) versus positive cases with strong contact (Hi-C ≥ 0.002). These weak positive cases did not follow the trend of regular enhancers in that they did not have strong Hi-C contact between the EP and the enhancer had lower activity (Fig. 7a and b). There was significant increase in both the TSS count and the remaining TSS contact from the focal enhancer, as well as the enhancer count, and the remaining enhancer contact to the focal TSS (Fig. 7c–f) among the positives with weak contact. This showed that the weak-contact positives tend to reside in high-density region of enhancers and promoters. The full results of different features are found in Supplementary Table S9.

Among the 601 positive EPs in Gasperini2019, there were 118, about one fifth of positive cases that have Hi-C contact <0.002. Of those, 72 positive EPs had an ABC score <0.0021 (Supplementary Table S10). We observed clustering in close genomic distance, as multiple positive cases around ATG2A on chromosome 11 or the Histone 1 cluster on chromosome 6. Some of them could be explained by an enhancer with unusually high activity compensating for the low contact as in the case of ATG2A. There is a possibility that the rest of them are erroneous calls due to variation in measurements, or an indirect effect through trans-acting regulation. But, given the clustering or adjacency we observed in this list, and the fact that a lot of these genes are known to be regulated by super-enhancers according to dbSuper (Hnisz *et al.* 2013), we think these are indeed functional, but atypical EP pairs that function through a different mechanism that relies on the presence of other enhancers or promoters in the genomic neighborhood. A model where enhancers act multiplicatively to regulate a single gene was recently proposed (Zhou *et al.* 2023), and could potentially explain such pattern where a weak enhancer is functional near a stronger enhancer.

## 4 Discussion

Since the supervised XGB model included components of the ABC score as input, and used the ABC software to generate many feature values, it is expected that XGB will outperform against the unsupervised model of ABC in this biased comparison. Thus, the performance comparison was not the end goal of the study, rather we hoped to learn additional insights about the problem through the model. But we also found that improved performance through the supervised approach was not guaranteed, especially if the test dataset has a distributional shift compared to the training data, through various targeting or filtering.

Given the nature of the experiment, what distinguished the positives and negatives were not only functional versus nonfunctional EPs, but also detectable versus not detectable by the experiment. Here, detectability had to do with both the power of the experiment, as can be seen with the importance of *TargetGeneExpression*, as well as robustness of the biological system against perturbation. As such, in addition to learning to distinguish EPs with low power, what the algorithm was learning to predict the negative cases was, somewhat unintuitively, the characters of a stronger or more robust activation that is unaffected by the perturbation. There was information to be gained in predicting the negatives, but it was different from what we naively expected.

For example, in XGB, the correlated features of strong activating marks H3K27ac and H3K4me3 and EMSY, HCFC1 peaks at the TSS led to fewer functional enhancers for that target TSS. This is consistent with the recent report that showed promoters of housekeeping genes called P2s with activating motifs had decreased responsiveness to distal enhancers (Bergman *et al.* 2022). In fact, the ChIP-seq peaks of HCFC1 and EMSY were identified in the study as part of the distinguishing features of P2 promoters (Bergman *et al.* 2022). We show that this pattern is replicated in a different experiment using CRISPR interference, compared to the massively parallel reporter assay used in Bergman *et al.* (2022). Similarly, the ChIP-seq peaks of the AP-1 complex at the enhancer were associated with negative predictions. Our interpretation of this pattern is that AP-1 bound enhancers are more robust, due to redundancy associated with enhancer hubs, and less likely to show effect of perturbation.

In our first draft, we missed several TFs that are well-known to bind to enhancers, and are routinely used to identify candidate enhancers, such as co-activators CREBBP (CBP), EP300 (P300), BRD4, and CEBPB. This was because the SHAP analysis was underestimating the contributions of these TFs, by spreading their contributions due to their collinearity. This unintended effect was more severe with co-activators that bind broadly across the genome and have many partners in co-binding. Once we reduced the dimensions with NMF and clustered the correlated TFs, we could see the clusters with these TFs as members (*TF_NMF4_e*, *TF_NMF6_TSS, TF_NMF5_TSS, TF_NMF7_e*, *TF_NMF8_e*) rising in importance ranks.

Largely, the TFs that contribute to predicting positive EP pairs were what we expected given our model cell line, TFs that are important for hematopoiesis and/or cancer. The only TF that showed up as important for prediction that is relatively less studied in the context of hematopoiesis or cancer was CHAMP1 (Supplementary Fig. S5), a TF associated with a neurodevelopmental disorder (Itoh *et al.* 2011, Hempel *et al.* 2015). CHAMP1 interacts with Rev7, HP1, and POGZ and functions in chromosome segregation and DNA repair (Li *et al.* 2022). We hypothesize that CHAMP1 is involved in protecting the chromosome integrity under replication transcription conflict. In addition to CHAMP1, there were several DNA repair proteins that showed up as an important predictor, including RAD51 as member of the *TF_NMF8_TSS* cluster, and XRCC3 at the TSS (Supplementary Fig. S5). Double-strand breaks (DSBs) are enriched at chromatin loops that mediate promoter–enhancer interaction with high transcriptional activity (Gothe *et al.* 2019). RAD51 is one example of a DNA repair protein that is known to co-localize with super-enhancers to protect the DNA through transcription-coupled repair (Hazan *et al.* 2019).

The supervised XGB is limited by its model system which is the K562 cell line. A lot of the positive EPs in Gasperini2019 are associated with cell cycle genes, including the histone gene cluster that is partially driven by the indirect enhancers described above. Likewise, the DNA repair genes that are predictive of functional enhancers maybe a pattern that is only found in cancerous cells that are under high DNA replication stress. Since the increased performance relies on the TFs that are potentially cell type specific, the model may not achieve the same prediction accuracy on other cells or tissues. This is both the strength and weakness of the model, in that it becomes more accurate to the system, and loses generalizability.

Finally, by studying the genome-scale data, we found that our understanding of enhancer–target specificity is still quite limited. The performance we saw in targeted studies did not replicate in the genome-wide data, without various filtering strategies. This is largely due to low power, and the targeting and filtering are well-designed to bring out the main principles of regulation. But, it also highlights the complexity outside the confined data that warrants more study. Some of the False Positives may be due to the redundant and robust enhancer network that buffers the effect of perturbation. In this case, the features around the enhancers that predict negative EPs, such as the AP-1 complex, could be a clue for further investigation. The False Negatives was found to increase with the overall enhancer and promoter density of the region. We highlighted a class of atypical False Negative enhancers that were not in direct contact with the promoter measured by Hi-C. Unlike other positive enhancers, these were more likely to have an effect in a high-density region where there were other strong/numerous enhancers and promoters nearby. Both the False Positives and the False Negatives identified in the genome-wide data suggest that the effect of other enhancers nearby could be important in resolving the discrepancy between the model and the experimental data.

## Supplementary Material

btae367_Supplementary_Data
