# Supplementary material for: Supervised learning of enhancer–promoter specificity based on genome-wide perturbation studies highlights areas for improvement in learning
Source: Bioinformatics. 2024 Jun 13;40(6):btae367. doi: 10.1093/bioinformatics/btae367 (PMC11211214; doi:10.1093/bioinformatics/btae367)
Supplement: btae367_Supplementary_Data [file btae367_supplementary_data.zip › supplementary_materials.rev2.pdf]

1 Supplementary Methods

1.1 Candidate Enhancer-promoter pair identification

To identify the potential candidate enhancer-promoter pairs in regulatory relationships, we decided to utilize the ABC software [1] that will identify all enhancer and target promoter pairs within a 5Mbp window for every gene in the genome. Briefly, the ABC software defines a list of 500 bp candidate enhancers centered around DHS peaks, and filters out enhancers overlapping an annotated Transcription Start Sites (TSS) as promoters. We consider the terms TSS and promoter as interchangeable throughout this study. For candidate enhancers that will be mapped to Gasperini2019, we used the DNase data *ENCFF001UWQ* and *ENCFF000SVO* from ENCODE following [2]. For candidate enhancers that will be mapped to Fulco2019, we used the DNase data *wgEncodeUwDnaseK562AlnRep1* and *wgEncodeUwDnaseK562AlnRep2* from the UCSC genome browser. For candidate TSS we used the collapsed promoter annotation file *RefSeqCurated.170308.bed.CollapsedGeneBounds.bed* provided by Fulco et al. as part of the ABC software [1]. We only include the genes considered to be expressed in the K562 cell line based on the RNAseq gene quantification data *ENCFF934YBO* from ENCODE.

1.2 Feature Engineering

The input to the machine learning pipeline consists of a feature matrix whose rows are enhancer-promoter pairs, and whose columns represent the variables measured for each entry. The features (variables) include measures of histone modification (H3K27ac, H3K4me3, H3K27me3) around the enhancer, the same histone modifications around the TSS, expression level of the target gene, genomic distance between the enhancer and the TSS, Hi-C contact measure between the enhancer and the TSS, the various TF presence at the enhancer measured from 250 ChIP-seq experiments from ENCODE, the 250 TF presence at the TSS.

The histone modification peaks were obtained from the histone modification ChIP-seq data from ENCODE (*ENCFF384ZZM* for H3K27ac, *ENCFF681JQI* for H3K4me3, *ENCFF937NEW* for H3K27me3) and processed following the approach of Fulco et al. The enhancer activity is quantified based on a combination (geometric mean) of DNase-seq and H3K27ac ChIP–seq signals following the approach of Fulco et al. [1].

We obtained the Hi-C scores for K562 generated from the data of Rao and Huntley et al. [3] using Juicebox dump with the hic matrix (*https://hicfiles.s3.amazonaws.com/hiseq/k562/in-situ/combined_30.hic*) provided by Aiden lab [4]. The hic matrices were processed following the approach of Fulco et al.[1]. Hi-C contact scores were calculated based on the KR normalized Hi-C signal between the enhancer and the TSS of the gene. We note here that although we relied on the ABC software for the candidate enhancer list and the normalization of histone modification marks and Hi-C intensities, we didn't use the final ABC score in our analysis, because we were interested in how individual features contributed to the prediction.

The transcription factor peaks were obtained from the "Transcription Factor ChIP-seq Clusters from ENCODE 3" track from the UCSC genome browser, summarized in a downloadable bed format at *https://hgdownload-test.gi.ucsc.edu/goldenPath/hg19/encRegTfbsClustered/encRegTfbsClusteredWithCells.hg19.bed.gz*. We extracted the peaks for the K562 cell type from the file and used bedtools to intersect the peaks of each TF with the set of all enhancers and promoters with the requirement that either 30% of the TF peak is covered or 30% of the enhancer promoter range is covered (*-e -f 0.3 -F 0.3*). The peaks were encoded into integers indicating the number of peaks present within the 500bp region. This process culminated in a set of 542 features for 65,713 enhancer-promoter pairs based on a set of 250 TFs. The full list of features can be found in Supplementary Table S1.

1.3 Training data, validation data and Independent test data.

To compile experimentally verified positive and negative enhancer-TSS pairs that we used for training, validation and test, we overlapped our candidate enhancers and promoters with experimental data from independent CRISPR perturbation studies, Gasperini2019 [2], Fulco2019 [1] and Schraivogel2020 [5]. For Gasperini2019, we used the file *Gasperini2019.at_scale_screen.cand_enhancer_x_exprsd_genes.200503.csv* found on their github repository *https://github.com/shendurelab/tafka-crisprQTL*. For Fulco2019, we used Supplementary Table 6a. of the publication. For Schraivogel2020, we used the file *etpa_chr8_chr11.input.txt* found at *https://github.com/argschwind/TAPseq*. The output we aimed to predict is a binary class label that was assigned based on the experiments in each study that is positive (equal to 1) only if blocking said enhancer using a CRISPRi gRNA resulted in significant (FDR<0.1) down regulation of the gene. The parameters for the bedtools intersect command (*bedtools intersect -wo -e -f 0.3 -F 0.3*) were determined based on the fraction parameter that gave us the maximum number of common enhancers between our candidate enhancers and Gasperini2019.

With Gasperini2019, the EP pairs that have 'NA' in the column 'pValueAdjusted' were filtered out. Within Gasperini2019, some of the enhancers were split into smaller segments targeted by different guide RNA, so there were a few *n:*1 relationships between the *n* smaller Gasperini2019 enhancers all contained within a single larger (500bp) candidate enhancer extracted from DHS peaks. For these, if any of the *n* enhancers were positive in the experiment, the whole candidate enhancer was called positive in our compiled data. With Fulco2019, we excluded the distal-promoter-gene pairs as described in the paper. Within Fulco2019, a few of the enhancers were combined and merged in their final table, leading to longer than expected enhancers. This resulted in both *n*:1 and 1:*n* relationships between enhancers tested in the experiment, and our candidate enhancers. In case of *n*:1, we followed the same rule, and considered our candidate enhancer positive if any of the contained enhancers tested were positive. In case of 1:*n*, all of our candidate enhancers were retained in our dataset, but they were all assigned the same positive or negative outcome based on the Fulco2019 results. With Schraivogel2020, we filtered the EPs to keep genes that have at least 1 positive enhancer, and less than 300 kb apart as described in the paper.

The compiled dataset of Gasperini2019 and the associated features was divided into training and test data, by setting aside chromosomes 5, 10, 15, and 20 as test sets. This resulted in a total of 29,291 EP pairs for training, and 4,274 EP pairs for test. Independent test datasets were generated by compiling the data from Fulco2019, Schraivogel2020 and the associated features in the same manner. The remaining training data of Gasperini2019 (chromosomes other than 5, 10, 15, 20) was then divided into a 4 x 4 nested blocked cross validation scheme (using *StratifiedGroupKFold*), where blocking was based on the groups defined by at least 5Mb of gap separating any of the enhancers or TSS in the data. Any enhancer-TSS pairs that are within the block were assigned to the same group, and all data within the same group were assigned into a single fold, ensuring separation by genomic distance, and thus independence between data across each fold [6,7]. The grouping of the data and folds can be found in Supplementary Table 2. The outer folds were used for evaluation of performance, while the inner folds were used for hyperparameter optimization.

1.4 Machine learning pipeline

We used gradient boosting (XGBoost) for the learning algorithm [8]. We explored other algorithms that didn't give us similar or better performance, so we don't report those results. As described above, the outer folds were used for evaluation of performance, so all of the scaling, feature selection, hyper-parameter optimization was done within the training split of the outer fold. Test split of the outer fold was used for unseen performance evaluation for the pipeline

trained on the train split. We didn't retrain the final pipeline on combined folds. We opted to evaluate the 4 different pipelines produced by 4 folds separately, to get a measure of uncertainty around our performance evaluation.

Within each train split, we scaled the features using MinMaxScaler to be between 0 and 1. We did feature selection using Boruta with SHAP values as importance measures on gradient boosted trees trained through a preliminary optimization [9]. We were more lenient in retaining the features by adjusting the max shadow features importance measure by 80%, and using a p-value cutoff of 0.1. The hyperparameters of the XGBoost classifier were optimized on the inner CVs using Optuna [10], with pruning and early stopping based on the validation set metric. Due to the extreme imbalance in our data (601/33,565 positive cases), we used Mean Average Precision (MAP) averaged across the inner folds as the metric to maximize during CV. We used the default algorithm of Tree-structured Parzen Estimator, and the MedianPruner on the validation MAP. The search space we explored and the final optimized parameters can be found in Supplementary Table 3.

The SHAP values were calculated using the tree SHAP implementation in XGBoost. SHAP values for the four test folds from the four models were combined through concatenation.

1.5 Chromatin Interaction Network (ChIN) generation

We generated a chromatin interaction network based on Hi-C contact maps centered around the promoter for each gene, where each node was an enhancer or promoter identified by the ABC software above, and the edge weights of the network were equal to the KR-normalized Hi-C contact between those nodes. At this stage, each gene has a very dense network that is both computationally inefficient to traverse and expensive to store in memory (Supplementary Fig. 1A). For this reason, we decided to simplify the network with edges that correspond to detectable chromatin loops. We used FitHiC2 [11] with a cutoff of FDR<0.01 to call significant loops, and only kept the contact edges corresponding to loops, filtering out all edges without a significant loop detected (Supplementary Fig. 1B). Once these simpler networks were created, they were further filtered by only keeping edges connecting enhancers and the promoters that were tested in Gasperini et al. [2]. From these simplified networks, the degree of each enhancer with respect to each nearby promoter was determined following [12]. In short, enhancers that were directly connected to the promoter with a significant Hi-C loop were labeled e_1, and enhancers that were not connected to the promoter, but were connected to an e_1 were labeled e_2 and so on. Enhancers within the same Hi-C window as the promoters were

labeled e_0, and enhancers that were more than 3 degrees away from the promoter or were not part of that promoter's subcomponent of the graph were labeled e_{inf}.

2 Supplementary Results

2.1 Enhancer and ChIN characteristics

In total, we generated 7,396 ChIN centered around each gene. The average number of nodes is 7.4, but the average number of edges is 13.3. The average degree of promoters is 1.5, so therefore most edges are not connected to the promoter. (Supplementary Fig. S15A-B). Nevertheless, the majority of the promoters in our networks tend toward a closeness measure of 1, underscoring their centrality within the network (Supplementary Fig. S15C).

Out of 33,565 EP pairs that were tested by Gasperini2019, 605 were positive, *i.e.* perturbation of the enhancer was found to significantly down-regulate the expression of the target gene. Of those 605 positive EP pairs, 26 were classified as e_0, 347 as e_1, 29 as e_2, 11 as e_3, and the remaining 192 as e_{inf}. An example of an e_2 is shown in Supplementary Fig. S1C. As expected, the further away from the promoter on the network, the lower the contact with the promoter (Supplementary Fig. S15D-E).

2.2 Reproducing the performance on Fulco 2019 data

There was some discrepancy between the performance of ABC reported in the original Fulco2019 and our initial results. We investigated the discrepancy and realized that Supplementary Table 6 contained both distal enhancer – gene pairs (DE-G) and distal promoter – gene (DP-G) pairs. Fulco did not include DP-Gs in their performance evaluation. Once we removed all DP-G pairs from the list, the ABC performance jumped significantly to what was reported in the paper.

Supplementary Figure S16 shows the improving performance of both ABC and XGB as DP-G interactions are removed.

2.3 Comparison with earlier studies

There are several earlier references that used the same dataset Gasperini2019 for enhancer promoter prediction. We compared the application of XGB and ABC score to the earlier studies. We found that in all of the references that use

the data Gasperini2019, there was discrepancy in the results coming from the different EP pairs present or absent in each dataset, even when we confirmed that the ABC score was very correlated (Supplementary Figure S18a).

With Schraivogel2020, there were a total of 2,334 EP pairs from Gasperini2019 that they considered in the evaluation after the filtering. Overlapping with the 33,565 EPs we compiled, we retrieved 1,891 EP pairs. The performance of XGB on this Gasperini dataset filtered by Schraivogel reached 0.64 (Supplementary Figure S17a). Schraivogel2020 also generated their own targeted perturbation data through the new approach called TAPseq [5]. We applied our XGB model on this dataset and achieved the performance of 0.45~0.51 which we show here again for comparison (Supplementary Fig. S17b). Schraivogel et al. used a Random Forest model to predict positive EPs learned on their targeted perturbation data of Schraivogel2020 and applied the learned model to the Gasperini2019 data, and vice versa. We reproduced this result using their model implementation and it is shown for comparison in Supplementary Fig. S17c.

Hecker et al. reported a total of 37,739 EP pairs of which 755 were considered positive for their application to Gasperini2019 (*Gasperini_interactions_wScore.txt*). When we overlapped the 33,565 EPs we obtained with Hecker2023, we retrieved 29,791 EPs. We confirmed that the calculation of ABC Score values were consistent between the two studies as the score themselves show a Pearson correlation of 0.9996 (Supplementary Figure S18a). Comparing the missing EPs, we noticed two important differences between our EP set and Hecker2023. 1) Hecker2023 had included the positive controls in Gaseprini2019, which were designed to target the TSS of genes, and thus are expected to be positive. 2) Hecker2023 used a significance threshold of adjusted p-value < 0.05 while Gasperini2019 and our study used adjusted p-value < 0.1. These two differences explained most of the discrepancy in the higher AUCPR for ABC scores they reported for Gasperini2019 and our lower AUCPR for ABC scores based on Gasperini2019. Other EPs that were missing were genes considered non-expressed in K562, that we do not consider in our analysis, or the EPs on chromosome X that we included but were excluded in Hecker2023. But these did not affect the performances. We applied the models to each subset of EPs using different criteria and show how the performance differs in Supplementary Figure S18. The initial performance on the EP pairs we recovered by intersecting our features with EP pairs from Hecker et al. (705/32831 positive rate) shows AUPRC of 0.32 for XGB and 0.48 for ABC (Supplementary Figure 18b). After loosening the significance threshold from <0.05 to <0.1 (988/32831 positive rate), AUPRC of XGB increases to 0.38 (Supplementary Figure 18c). After we remove the TSS targeting positive controls (593/30034 positive rate), AUPRC of XGB decreases to 0.33 and the AUPRC of ABC

score decreases to 0.21 (Supplementary Figure 18d), consistent with the performance we see in our main study (Figure 1).

We also show that once we only keep genes that have at least one positive enhancers, our performance jumps significantly to AUC that is higher (Figure 2). But, we also observe that, after filtering, additional information such as TFs are not any more informative compared to simpler models such as ABC.

2.4 Hi-C contact

The SHAP value for Hi-C contact, the most important feature, rose with a steep slope, indicating that Hi-C doesn't need to be very large in value to contribute. Even a KR-normalized Hi-C contact frequency as small as 0.01 increased positive prediction, and by the Hi-C value of 0.03 the SHAP values plateaued and showed large variance indicating influence of other features (Supplementary Fig. S5A-B). Since SHAP values are based on the trained predictive model, we explored whether the signal can be seen in the actual data itself. Data from Gasperini2019 (and Fulco2019) showed large differences in the Hi-C contact strengths between positive and negative pairs (Supplementary Fig. S5C). Similar to SHAP values, we saw an increase in the proportion of significant pairs as Hi-C value increased to as low as 0.01 and we observed the plateau by Hi-C contact value of 0.03. (Supplementary Fig. S5D). Among the functional EP pairs, the effect size showed larger negative values with stronger Hi-C contact (Supplementary Fig. S5E), meaning stronger down-regulation with CRISPR interference, *i.e.* stronger functional effect.

2.5 H3K27ac, H3K4me3, C11orf30 (EMSY) at the TSS, and HCFC1 (HCF1) at the TSS

The two most important TFs are C11orf30 (EMSY) at the TSS, and HCFC1 (HCF1) at the TSS, which were both associated with nonfunctional EP prediction (Supplementary Fig. S8,S9). Since EMSY and HCFC1 each interact with the *Sin3* histone deacetylase (HDAC) respectively [13], and EMSY is known as a repressor [14,15], we initially thought this is through the repressive action of EMSY and HCFC1 at the promoter. But, unexpectedly, Both EMSY and HCFC1 peak at the TSS were associated with higher H3K27ac marks and H3K4me3 marks at the TSS, and more active promoters (Supplementary Fig. S9A-D). This was also confirmed through the hierarchical clustering of SHAP values across features. The clustering shows that H3K27ac, H3K4me3, and HCFC1 at the TSS are correlated in their effect on prediction (Supplementary Fig. S9E). The correlation is also found in the ChIP-seq and chromatin data that

shows higher H3K27ac and H3K4me3 chromatin marks when either of the TFs are present. The ChIP-seq peaks between the two TFs are also co-occurring more often than by chance. Correlated features of H3K27ac, H3K4me3, HCFC1 and EMSY peaks at the TSS together lead to decreased prediction of functional enhancers affecting the target gene.

Many chromatin-associated proteins that were initially known to be either a repressor or an activator are revealing dual roles. EMSY was first characterized as a repressor [14] that interacts with BRCA2, but subsequent reports suggest EMSY can also activate transcription [15]. Likewise, HCFC1 is known to associate with both activator and repressor E2F proteins in different stages of the cell cycle regulation [16]. In our model, the correlated features of EMSY, HCFC1, H3K27ac and H3K4me3 at the TSS led to fewer functional enhancers for that target TSS. This is consistent with the recent report that showed promoters of housekeeping genes called P2s with activating motifs had decreased responsiveness to distal enhancers [17]. .

2.6 TFs at the enhancer

On the other hand, numerous transcription factors found at the enhancer region were predictive of the functional EP pairs in the positive direction. The most important TFs found are the well-known hematopoietic and/or cancer related TFs, STAT2, STAT1, CHAMP1, STAT5A, GATA2, FOXM1, etc. (Supplementary Fig. S10, S11)

The only TF that shows up as important for prediction that is relatively less studied in the context of hematopoiesis or cancer would be CHAMP1, a TF associated with a neurodevelopmental disorder [18,19]. CHAMP1 interacts with Rev7, HP1, and POGZ and functions in chromosome segregation and DNA repair [20]. CHAMP1 presence at the enhancer is correlated with FOXM1 presence at the enhancer (correlation coefficient 0.428) and they both increase the prediction of positive EP pairs. Given the function of the gene, it is possible that the gene is involved in protecting the DNA during replication transcription conflict, and functional enhancers are especially at risk for replication stress in this cancerous system.

2.7 Rest-of-ABC

In addition, the EP is more likely to be positive when the value of the rest-of-ABC score (ABC denominator – numerator) is lower, *i.e.* when the neighboring enhancers are weaker (although we see some interesting exceptions

that we describe below). This also justifies the approach of ABC, which compares the relative effect of each enhancer, normalized by the sum of ABC of other nearby elements.

One exception was the feature rest-of-ABC (ABC denominator-numerator) that showed a distinct pattern between the e1minus and e2plus EPs. Only in the e2plus EPs, we saw a subset of EP pairs that showed opposite trend from the rest of the data, *i.e.* stronger positive prediction when the rest-of-ABC is larger (Supplementary Fig. S19 B-C). The pattern is replicated in the whole data (Supplementary Fig. S19). The same subset of EP pairs shows an interesting pattern going against the trend with the H3K4me3 at the TSS as well (Supplementary Fig. S20). Generally, the H3K4me3 mark at the TSS shows good correlation with the H3K27ac mark at the TSS, but this particular pattern only showed up with H3K4me3, and not with H3K27ac (Supplementary Fig. S20 vs. Supplemenetary Fig. S6).

3 Supplementary Tables.

Supplementary Table S1. Full list of features before and after feature selection uploaded separately.

Supplementary Table S2. Grouping of EPs based on genomic distance used for block cross validation uploaded separately.

Supplementary Table S3. Final optimized hyperparameters uploaded separately.

Supplementary Table S4. Comparison with experimentally validated EPs in Gasperini et al. uploaded separately.

Supplementary Table S5. EP predictions exclusively correct for XGB vs. ABC.

Supplementary Table S6. Individual Trees learned with XGBoost uploaded separately.

Supplementary Table S7. Performance on highly expressed genes

Supplementary Table S8. Performance for EP e1minus vs. e2plus

Supplementary Table S9. Different features between positives with strong vs. weak contact uploaded separately.

Supplementary Table S10. Positive EPs with weak contact uploaded separately.

				ABC prediction	
				TRUE	FALSE
Significance	TRUE	XGB prediction	TRUE	387	35
			FALSE	35	144
	FALSE	XGB prediction	TRUE	1969	974
			FALSE	2395	27626

Supplementary Table S5. EP predictions exclusively correct for XGB vs. ABC. The counts of predictions are categorized based on the True Significance, and whether each model, XGB or ABC discovered it or missed it. There are 35 positives exclusively found by ABC and 35 positives exclusively found by XGB. There are 974 negatives exclusively found by ABC and 2395 negatives exclusively found by XGB.

Gasperini2019 Test (chr 5,10,15,20)					
model	data	aucpr	avg_precision	bal_accuracy	f1_score
model1	Gasperini Test	0.294	0.300	0.835	0.266
model2	Gasperini Test	0.275	0.284	0.833	0.250
model3	Gasperini Test	0.309	0.313	0.835	0.259
model4	Gasperini Test	0.325	0.328	0.832	0.231

Supplementary Table S7. Performance on highly expressed genes. The performance improves when we filter out the lowly expressed genes, and only keep the highly expressed with a threshold of TargetGeneExpression >= 2.5.

a.

		Outer Folds Test			
model	data	aucpr	avg_precision	bal_accuracy	f1_score
model1	fold1 testsplit	0.318	0.322	0.779	0.329
model2	fold2 testsplit	0.394	0.398	0.794	0.375
model3	fold3 testsplit	0.343	0.347	0.784	0.361
model4	fold4 testsplit	0.434	0.438	0.770	0.306

b.

		Outer Folds Test			
model	data	aucpr	avg_precision	bal_accuracy	f1_score
model1	fold1 testsplit	0.093	0.083	0.500	0.015
model2	fold2 testsplit	0.083	0.088	0.633	0.024
model3	fold3 testsplit	0.038	0.043	0.627	0.023
model4	fold4 testsplit	0.106	0.116	0.625	0.023

Supplementary Table S8. Model performance for model trained on EPs with vs without direct Hi-C contact. performance measure for models trained on A) EPs with direct contact (e1minus data) and B) EPs without direct contact (e2plus data).

4 Supplementary Figures

Supplementary Fig. S1. Chromatin Interaction Network Generation.

Supplementary Fig. S2. The effect of TF ChIP-seq quality on the performance

Supplementary Fig. S3. Enrichment of features in EPs exclusively identified by XGB and ABC.

Supplementary Fig. S4. Distribution of Target Gene Expression in Gasperini vs. Fulco

Supplementary Fig. S5. Top 100 features SHAP values

Supplementary Fig. S6. Hi-C

Supplementary Fig. S7. H3K27ac_TSS

Supplementary Fig. S8. Full NMF membership matrix for TSS and enhancers.

Supplementary Fig. S9. EMSY HCFC1

Supplementary Fig. S10. EMSY and HCFC1 correlation with H3K27ac

Supplementary Fig. S11. SHAP values without NMF clustered TFs.

Supplementary Fig. S12. STAT1 and STAT2

Supplementary Fig. S13. SHAP values for e1minus vs. e2plus

Supplementary Fig. S14. Remaining.contact for whole data

Supplementary Fig. S15. ChIN and enhancer characteristics.

Supplementary Fig. S16. Reproducing the performance of ABC Score on Fulco2019

Supplementary Fig. S17. Comparison with earlier study Schraivogel et al.

Supplementary Fig. S18. Comparison with earlier study Hecker et al.

Supplementary Fig. S19. Rest-of-ABC

Supplementary Fig. S20. H3K4me3 at TSS

Supplementary Figure 1

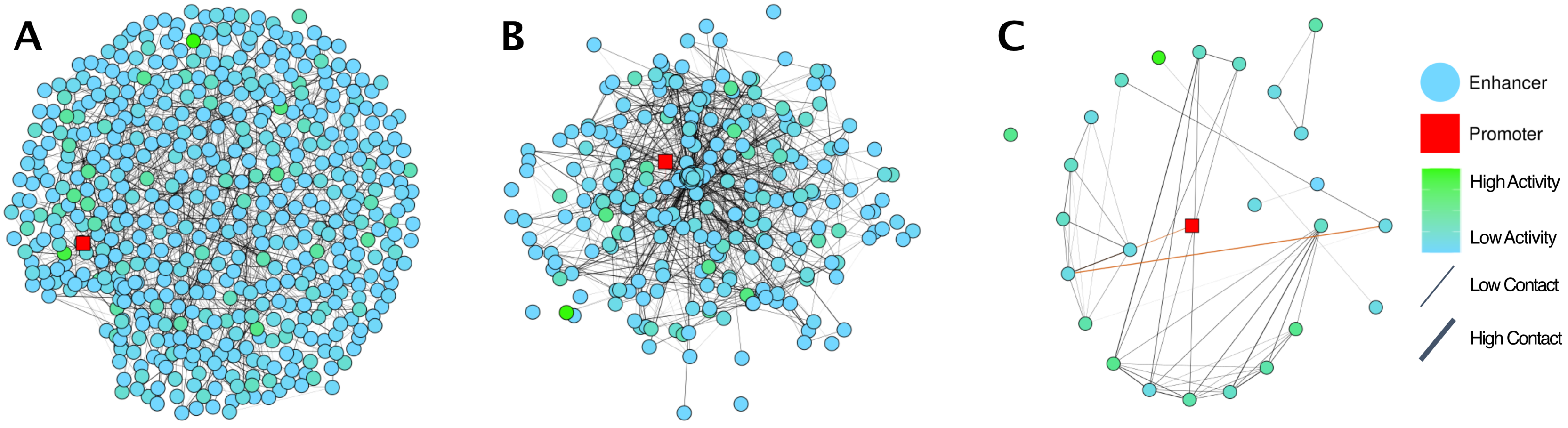

Supplementary Figure S1. Chromatin Interaction Network (ChIN) generation process. Chromatin Interaction Network generation process of the HIST1H2BD gene and its highly dense surrounding Hi-C contact network. Nodes are candidate enhancers identified based on open chromatin, and edges are the KR-normalized Hi-C contact scores between two nodes. The promoter is shown in red and enhancer nodes are color scaled based on their ABC activity score (green; high, blue; low). **A)** Unfiltered network showing all Hi-C contacts. **B)** Network with edges filtered based on significant Hi-C contact (FitHiC2 q-value < 0.01). **C)** Network with nodes filtered based on experimental data. Only experimentally tested (Gasperini et al., 2019) enhancer nodes are present in graph. The path connecting a significant e_2 enhancer to the promoter is highlighted in red.

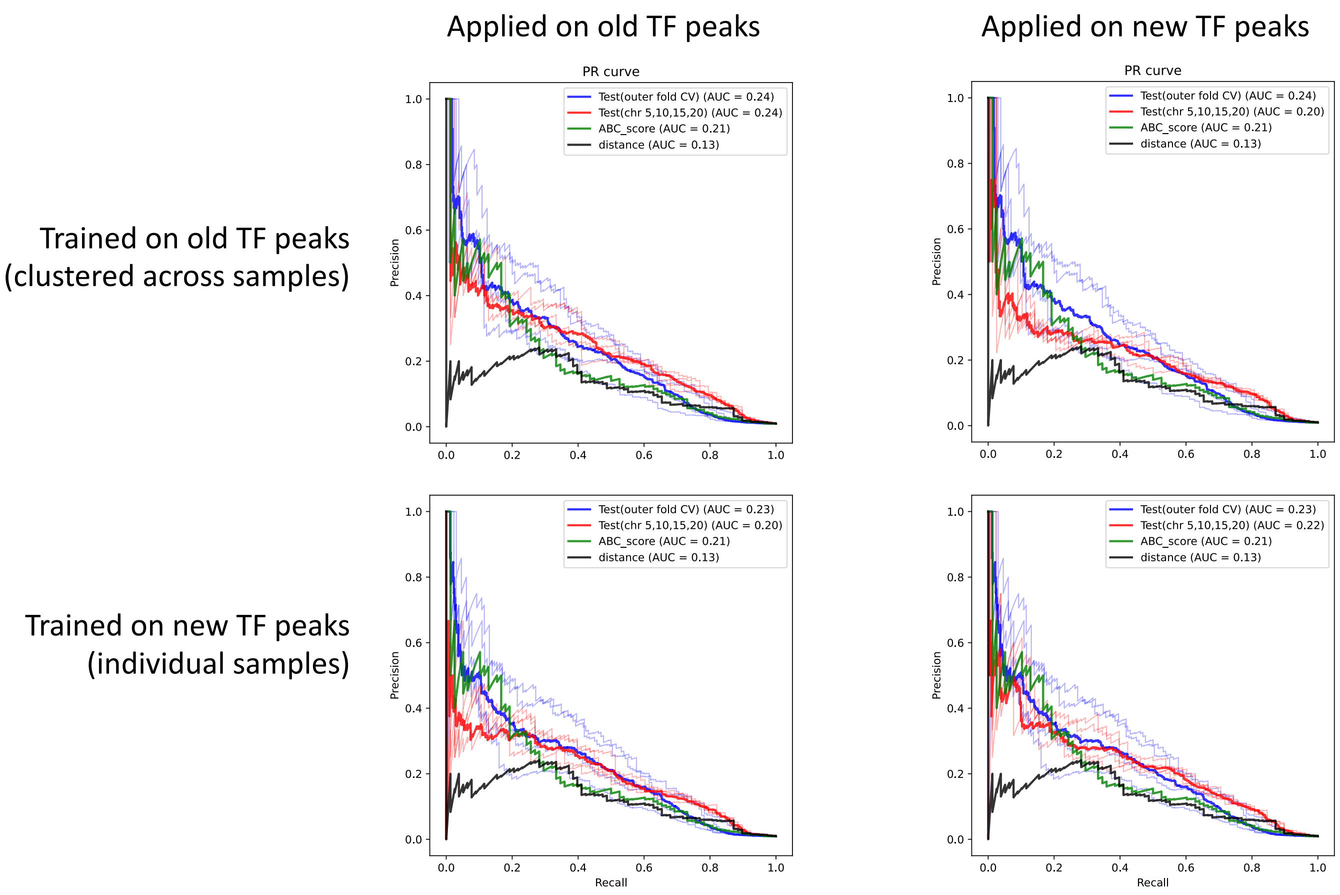

Supplementary Figure S2. The effect of TF ChIP-seq quality on the performance. PR Curves showing performance of the model when TF ChIP-seq features are replaced by a new dataset. There is slightly higher performance when we use the more conservative ChIP-seq peaks produced through clustering compared to the individual TF peaks. The larger difference in performance can be seen in the test performance (Chromosomes 5,10,5,20) when the model is trained on one set and applied on the other.

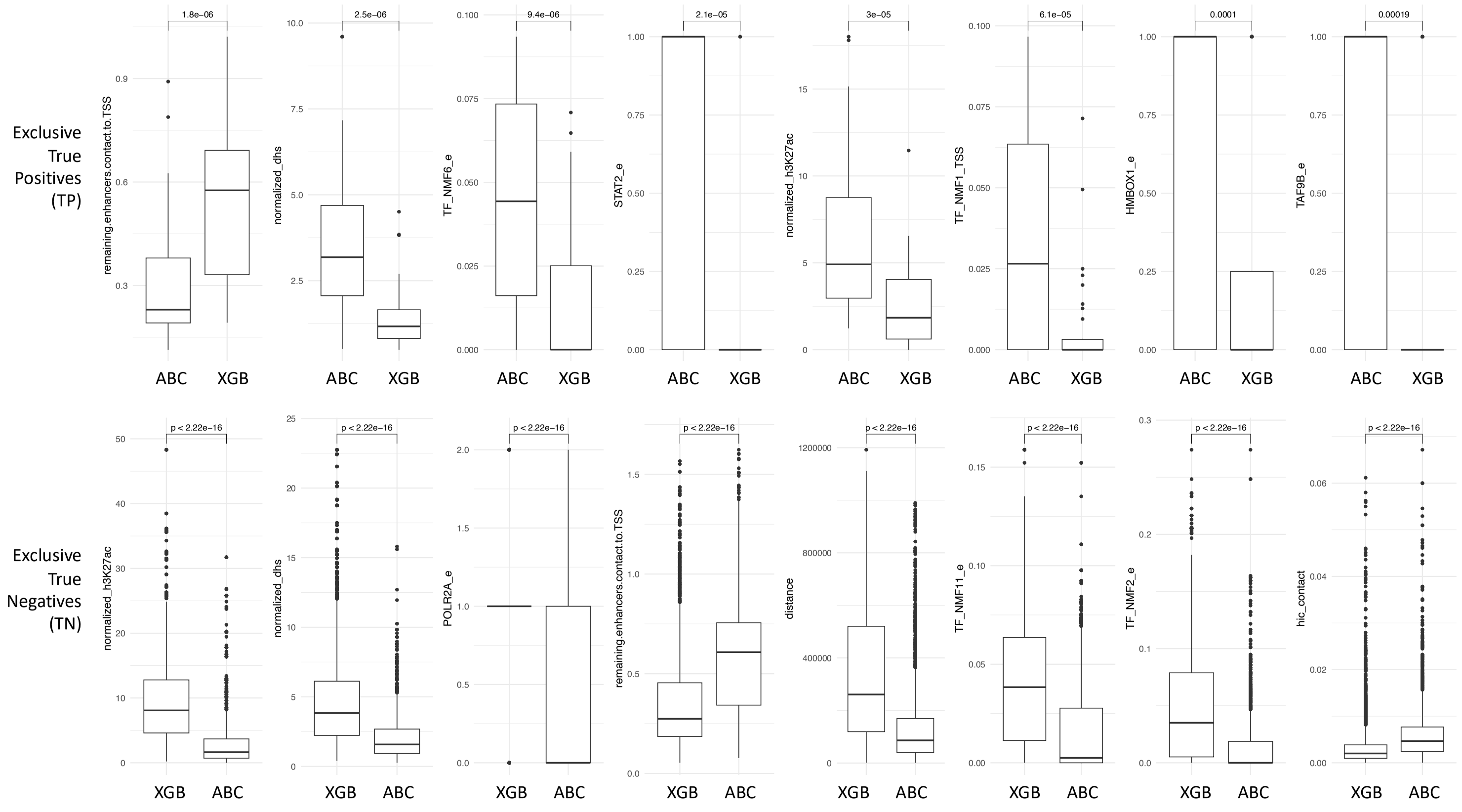

Supplementary Figure S3. Enrichment of features in EPs exclusively identified by XGB and ABC. The cases found as exclusive by each model (Supplementary Table S5) are compared for enrichment in features, including Histone and TF ChIP-seq.

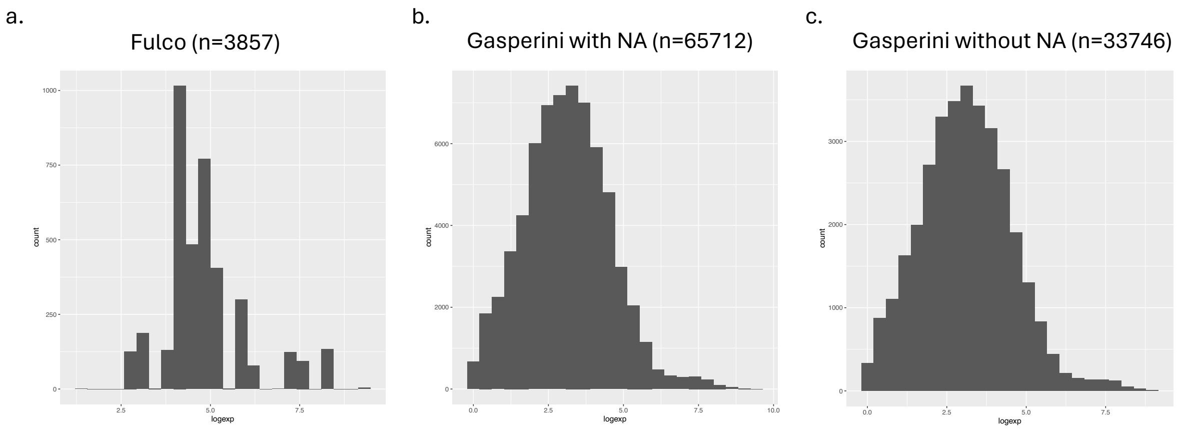

Supplementary Figure S4. Distribution of log-transformed Target Gene Expression in Gasperini vs. Fulco. Targeted experiments (Fulco2019) are designed to have genes with higher expression compared to genome-wide data (Gasperini2019).

Supplementary Figure S5. Top 100 features SHAP values uploaded separately.

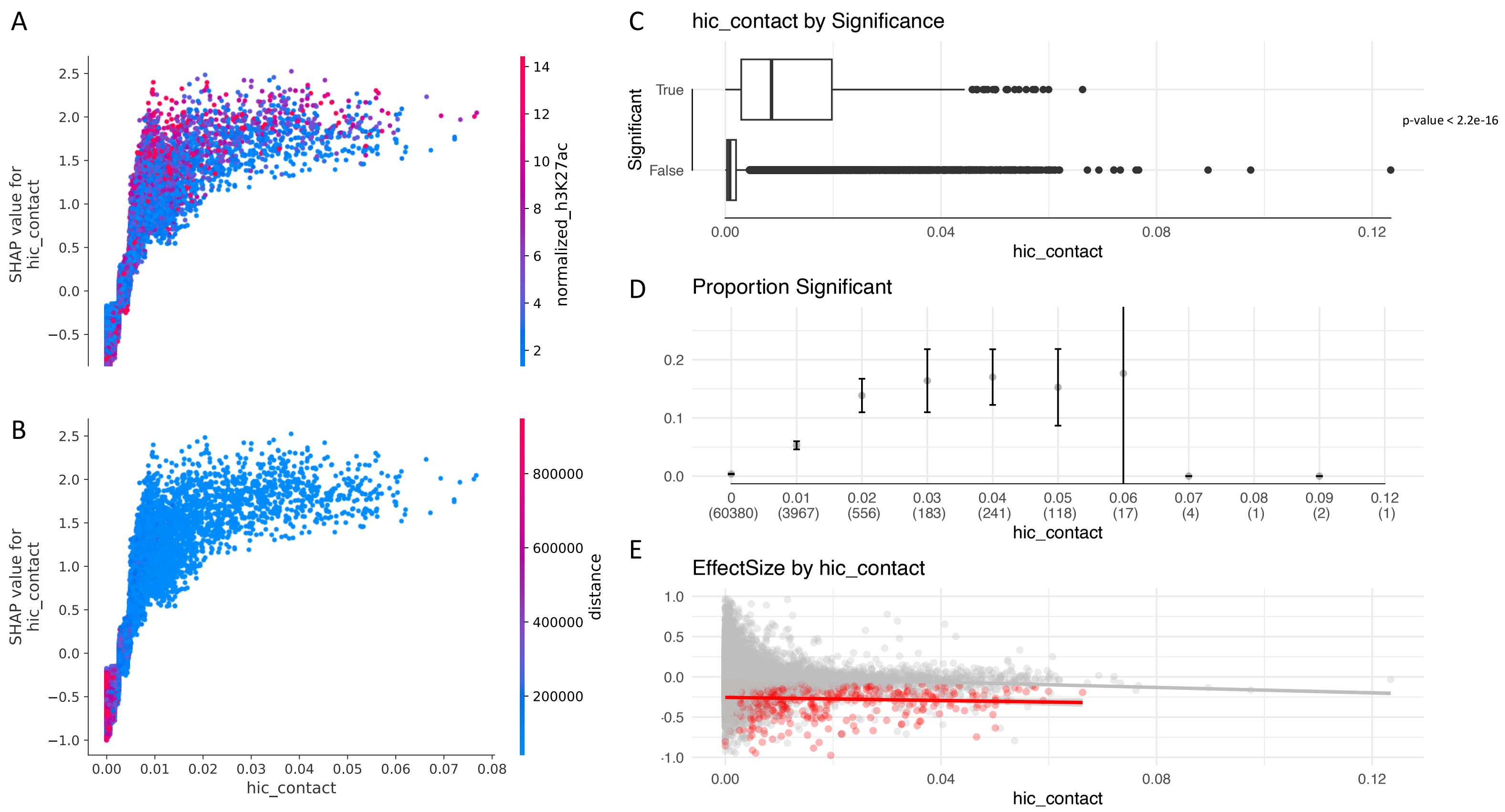

Supplementary Figure S6. Strong Hi-C contact predicts functional enhancer-promoter pairs. A) Hi-C contact shows positive trend with SHAP values, and interaction with H3K27ac at the enhancer. Vertical pattern of coloring for the same values of Hi-C contact reveals interaction between Hi-C and H3K27ac, recapturing the dynamics of the ABC model. **B)** SHAP values for Hi-C contact colored by genomic distance between enhancer and promoter. **C)** Contact by significance boxplot shows functional EPs are in strong Hi-C contact more often than nonfunctional EPs. **D)** The proportion of significant EPs increases as Hi-C contact increases. Numbers in parentheses show the number of samples (n) in each Hi-C bin. **E)** Effect size vs Hi-C score (significant samples in red). Significant EP pairs with stronger Hi-C contact have larger negative effect size (larger down-regulation from CRISPRi on paired enhancers).

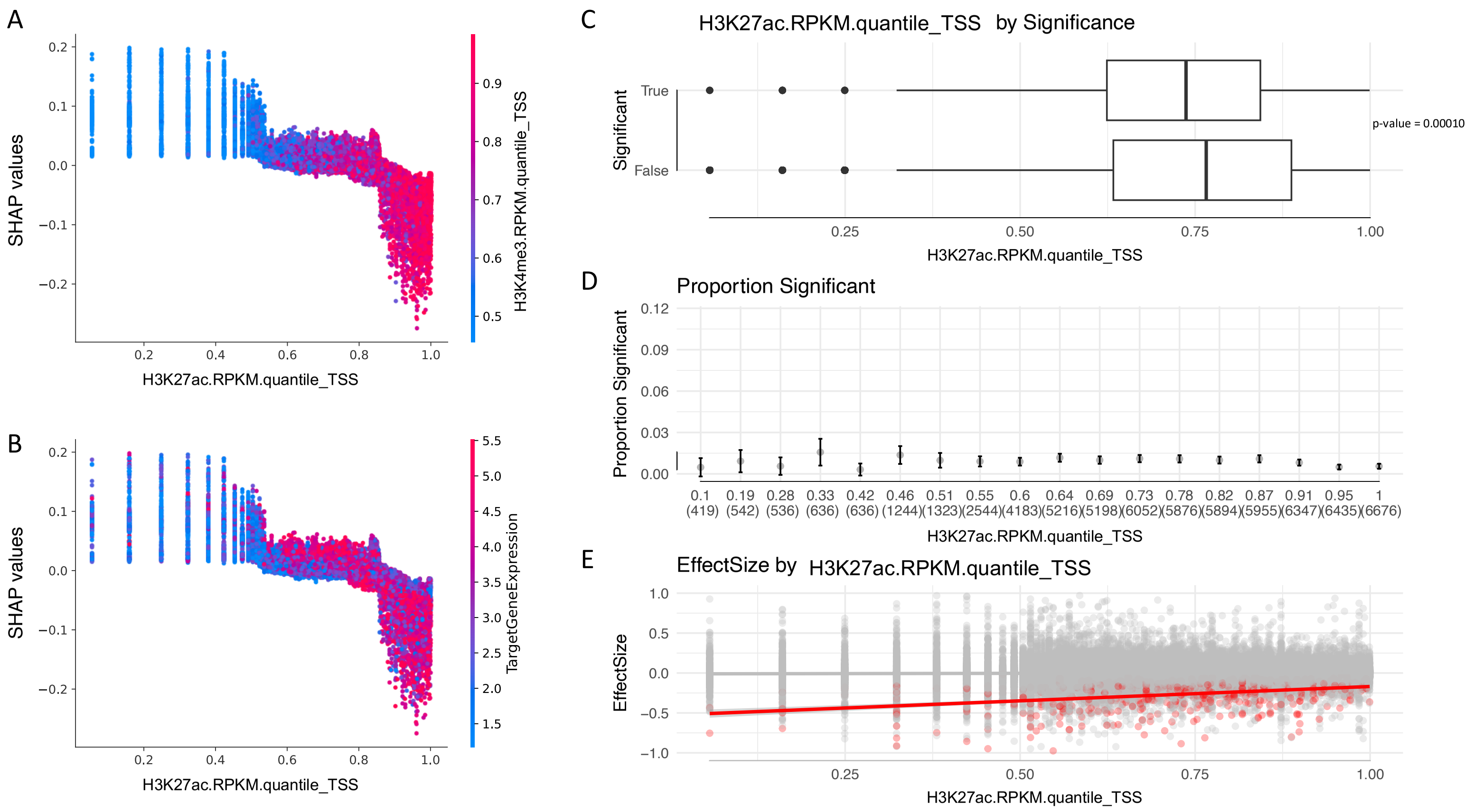

Supplementary Figure S7. Strong H3K27ac at the promoter predicts nonfunctional enhancer-promoter pairs. Promoter H3K27ac mark shows negative trend of SHAP values. Promoter H3K27ac shows correlation with **A)** promoter H3K4me3 and **B)** target gene expression. **C)** Promoter H3K27ac by significance boxplot. Significant EP pairs show decreased promoter H3K27ac . **D)** There is reduced significance among the target genes with the strongest H3K27ac marks at promoters. **E)** Effect size vs H3K27ac strength (significant in red). Strong H3K27ac promoters have smaller negative effect size (weaker down-regulation effects from CRISPRi on paired enhancers).

Supplementary Figure S8. Full NMF membership matrix for TSS and enhancers uploaded separately.

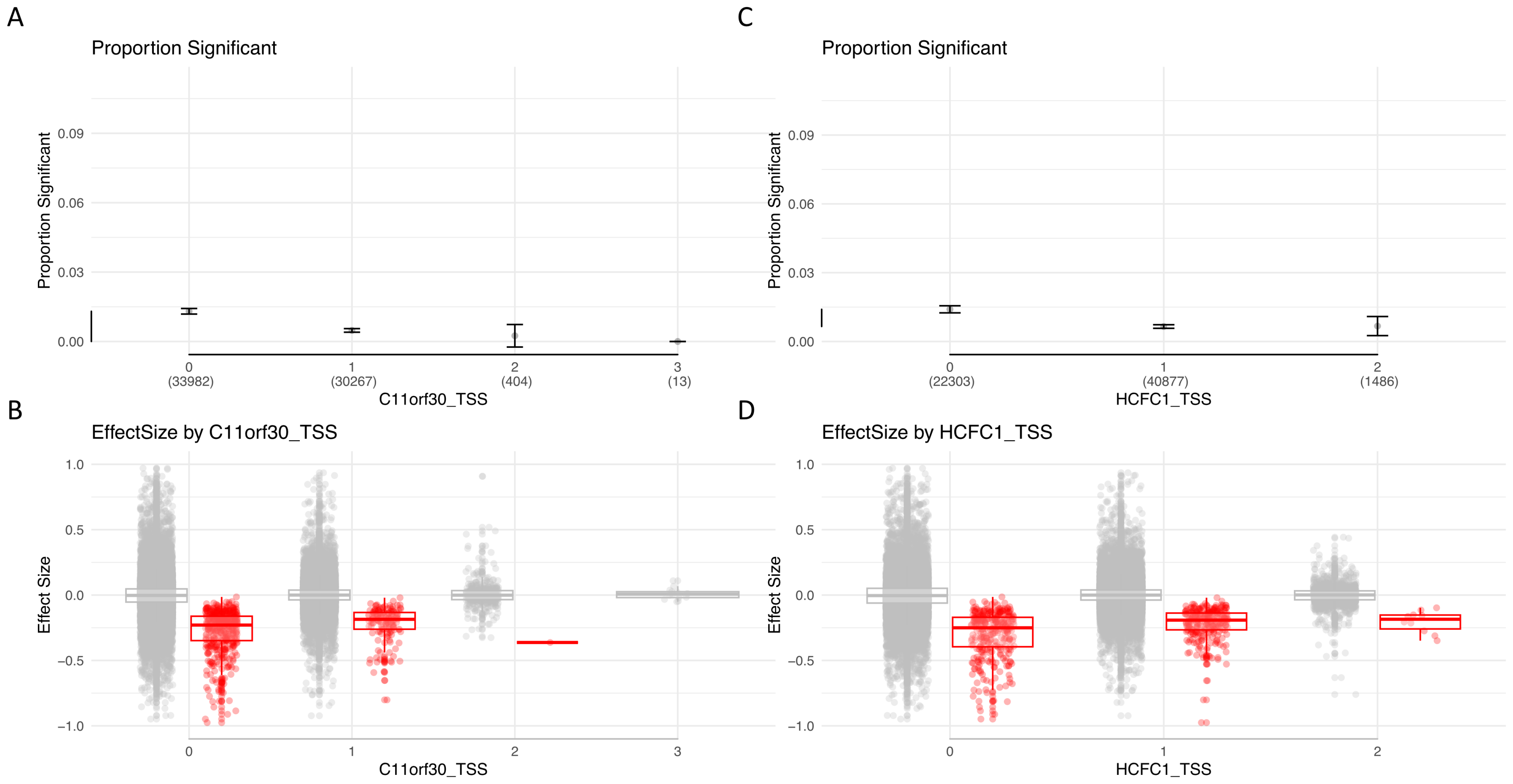

Supplementary Figure S9. EMSY(C11orf30) and HCFC1 at the promoter is associated with less significance and smaller effect size. A) Presence of ChIP-seq peaks of **A)** EMSY and **C)** HCFC1 found at promoters are associated with lower proportion of significant EP pairs. Effect size by the number of **B)** EMSY and **D)** HCFC1 at promoter. The presence of EMSY or HCFC1 at the promoter are associated with smaller negative effect size (weaker down-regulation effects from CRISPRi on paired enhancers).

Supplementary Figure 4

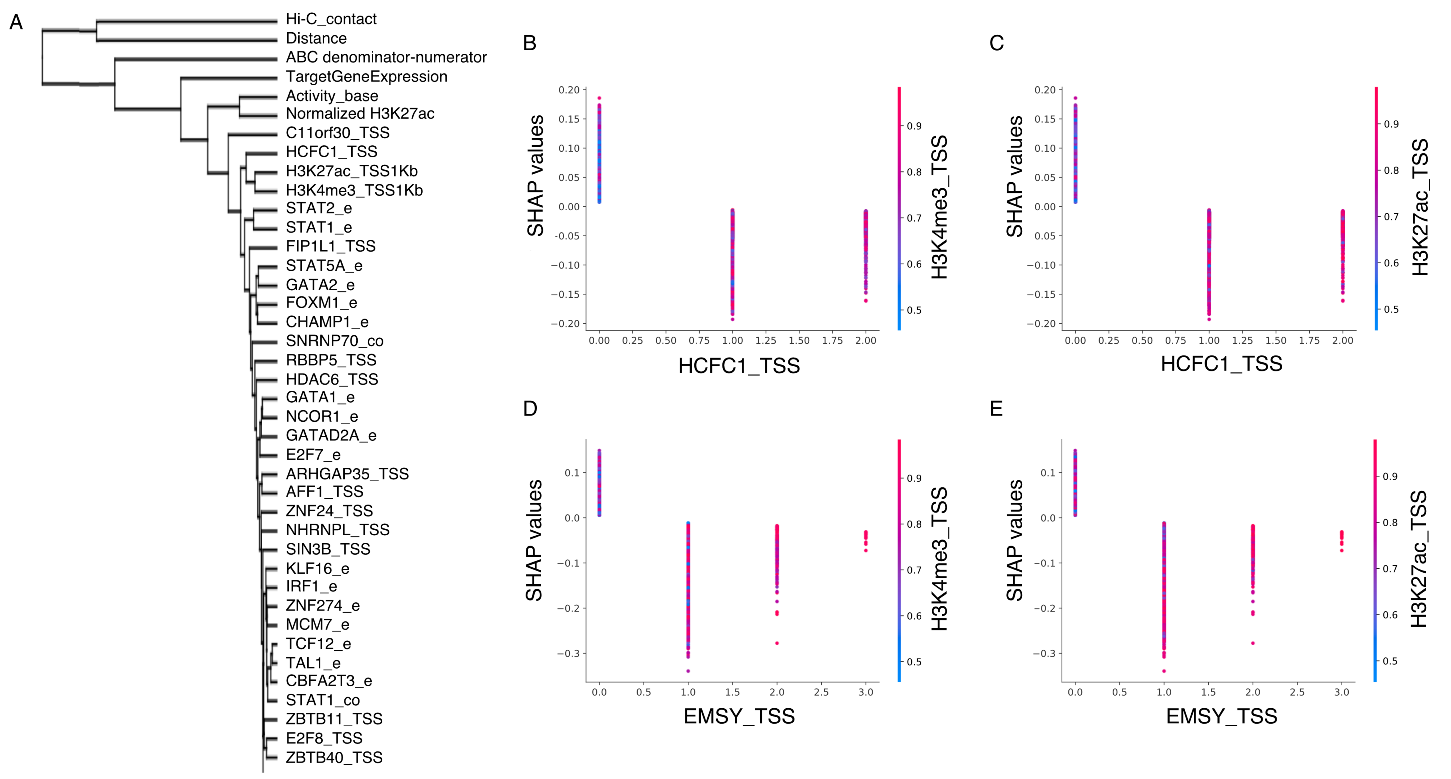

Supplementary Figure S10. EMSY(C11orf30) and HCFC1 at the promoter predicts nonfunctional enhancer-promoter pairs. A) Hierarchical clustering of SHAP features based on their contribution to prediction. HCFC1 at the TSS is correlated with H3K27ac and H3K4me3 at the TSS in predicting nonfunctional EP pairs. **B-E)** SHAP values of *HCFC1* and *EMSY* (*C11orf30*) at the promoters. Presence of *HCFC1* and *C11orf30* peaks show correlation with both H3K27ac and H3K4me3 marks at the promoters, and show lower SHAP values indicating prediction of nonfunctional EP pairs.

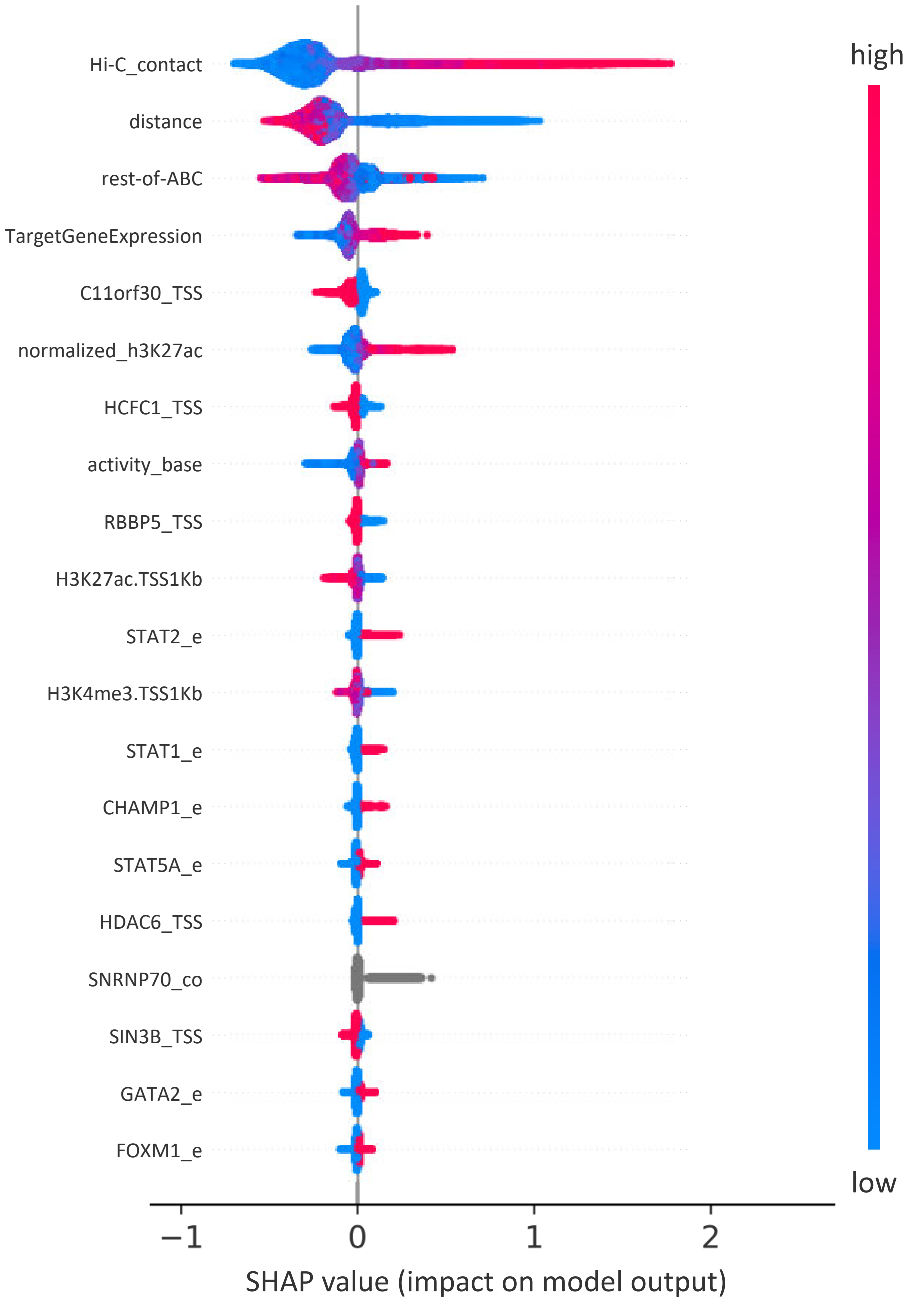

Supplementary Figure S11. SHAP values without NMF clustered TFs. Shap values for models trained without NMF clustered TFs show individual TFs that are important in prediction.

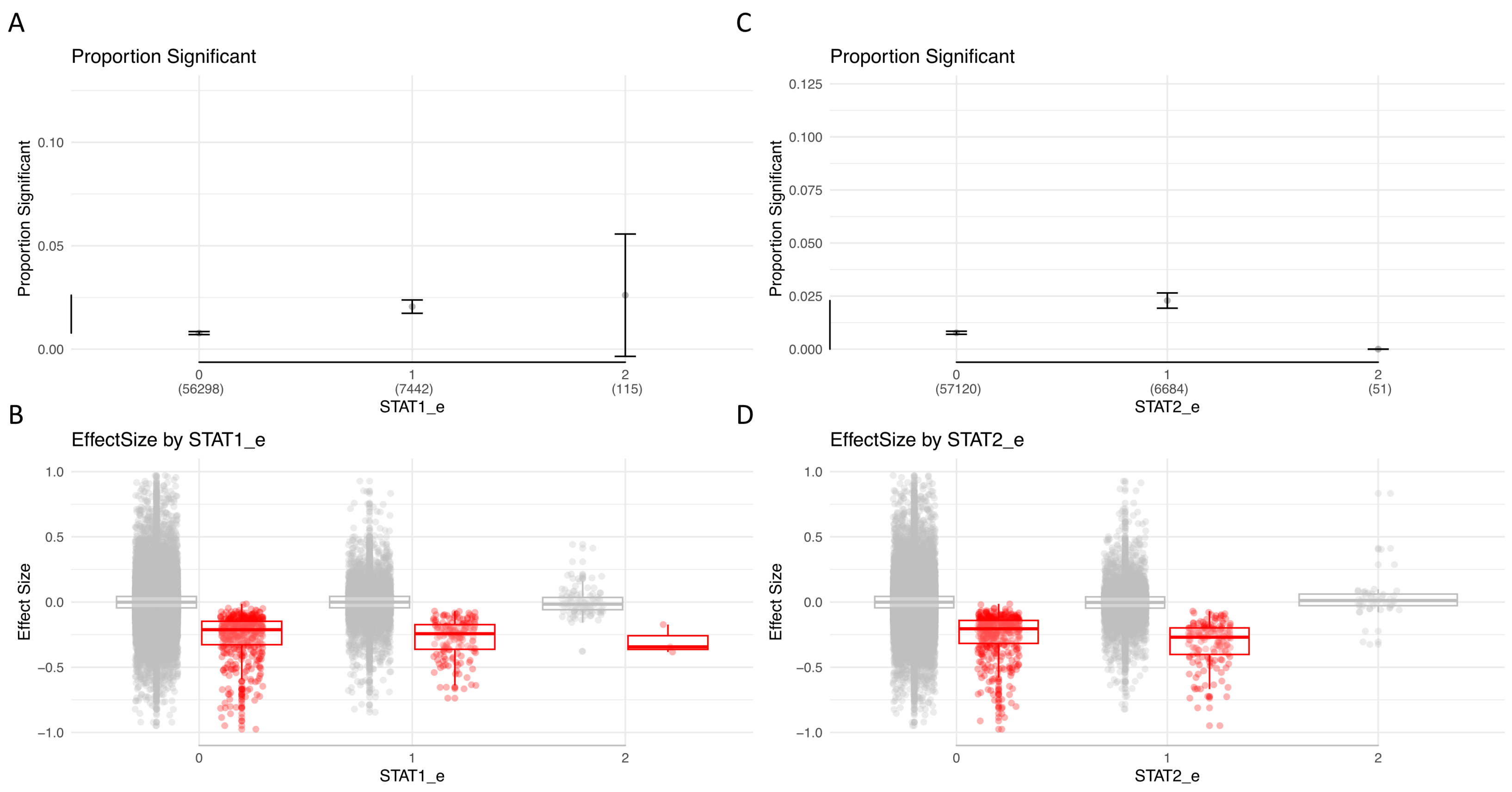

Supplementary Figure S12. STAT1 and STAT2 at the enhancer predicts functional enhancer-promoter pairs. Presence of ChIP-seq peaks of **A)** STAT1 and **C)** STAT2 found at enhancers are associated with higher proportion of significant EP pairs. Effect sizes by the number of **B)** STAT1 and **D)** STAT2 peaks at the enhancer. The presence of STAT1 or STAT2 at the enhancers are associated with larger negative effect size (stronger down-regulation effects from CRISPRi on the enhancers).

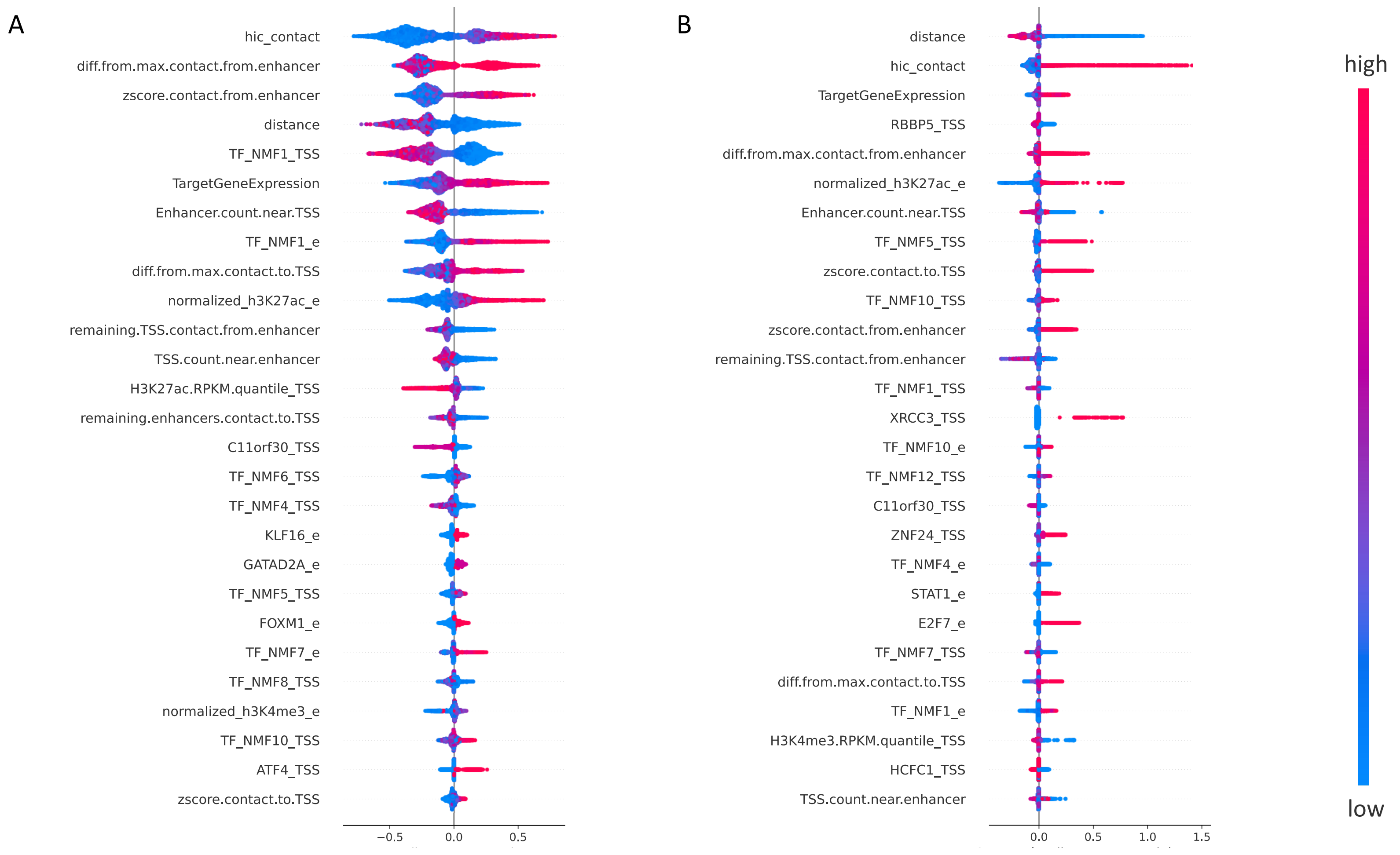

Supplementary Figure S13. **SHAP values for model trained on EPs with vs without direct Hi-C contact.** SHAP values showing important feature contributions for model trained on **A)** EPs with direct contact (e1minus data) and **B)** EPs without direct contact (e2plus data).

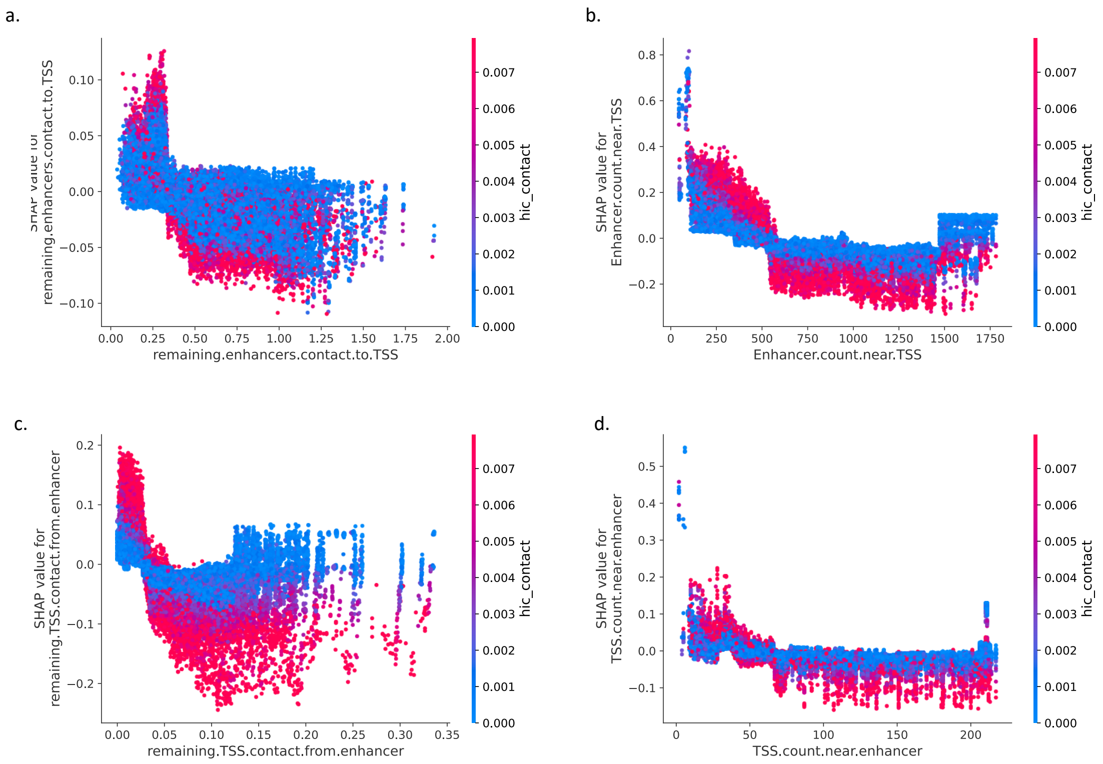

Supplementary Figure S14. SHAP values for features on regulatory element density. The pattern that it is more likely to predict positive when there are many/strong other contacts nearby for a subset of EPs found among indirect contact EPs are also observed in the model trained on whole data.

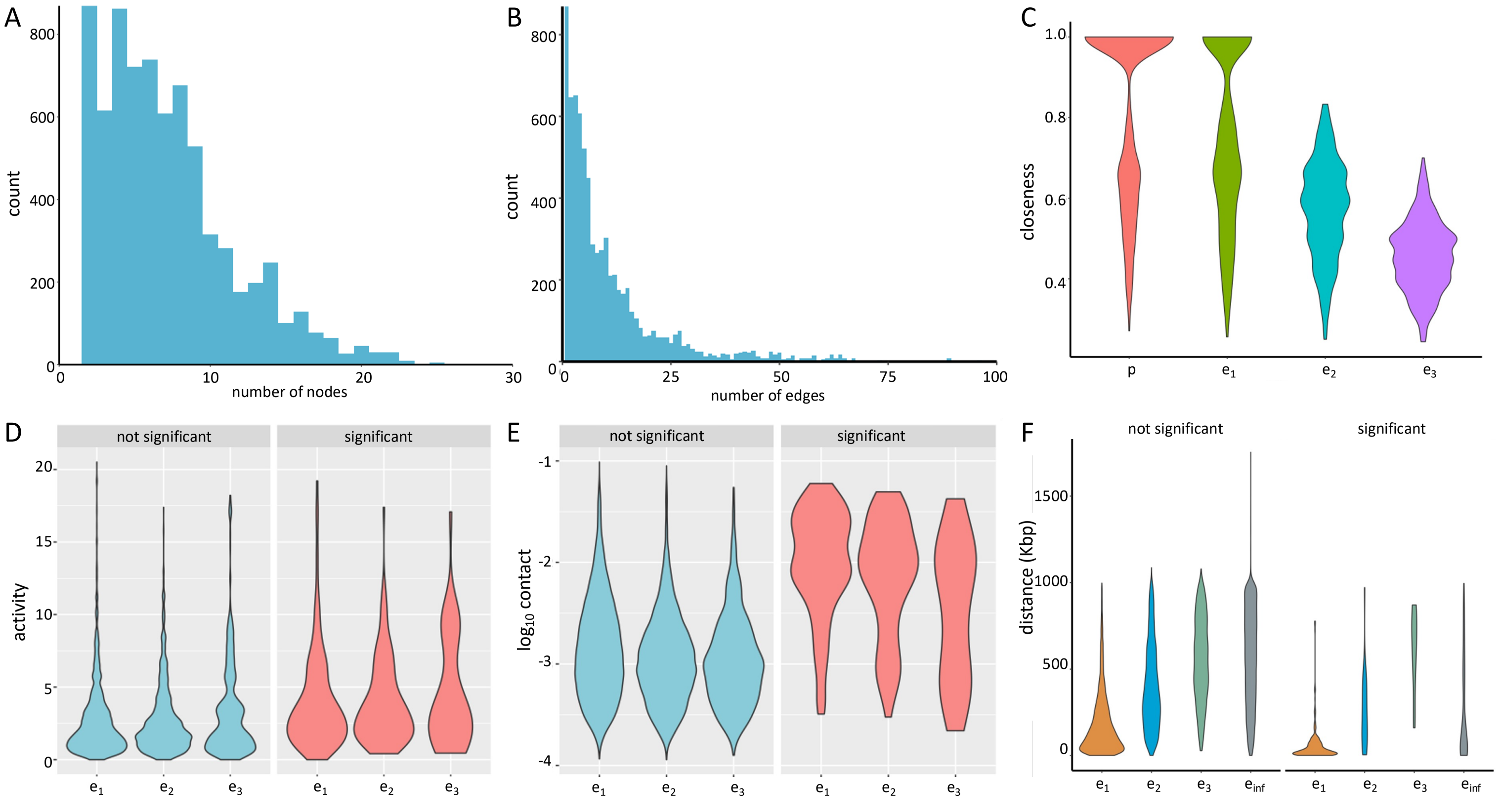

Supplementary Figure S15. ChIN and enhancer characteristics. Histograms showing distribution of the number of nodes **(A)** and edges **(B)** for each network. **C)** Closeness measure of each class, e_1 and promoter show higher closeness centrality to each network. Activity **(D)** and Contact **(E)** within each class of enhancer delimited by functional significance in Gasperini et al. 2019. **F)** Linear distance between each class of enhancer and their corresponding promoters measured in kbp.

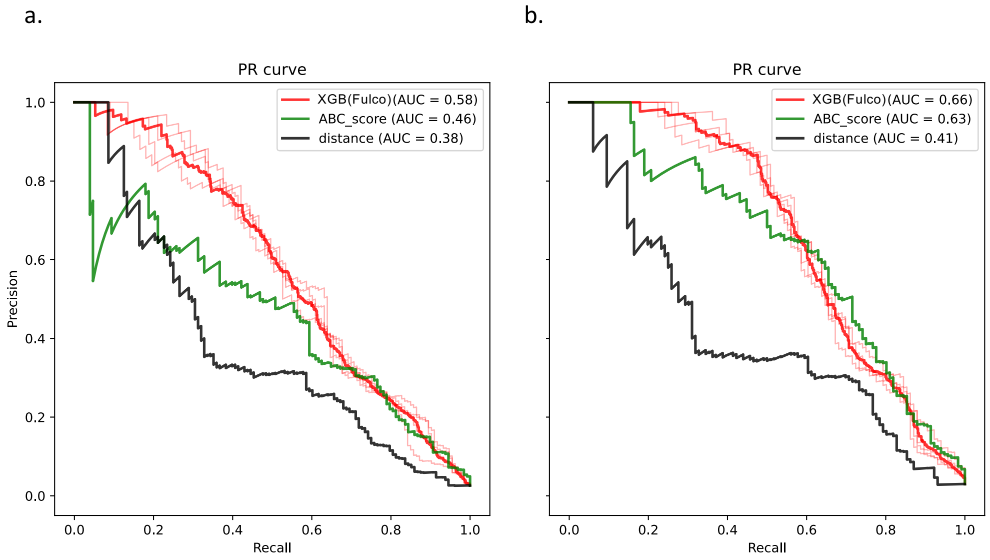

Supplementary Figure S16. Reproducing the performance of ABC Score on Fulco2019. The PR curves show improvement in performance with the Fulco2019 data for both ABC and XGB model, after filtering out the DP-G pairs from the data. a) Initial performance on total EP pairs. b) After removing the distal-promoter-gene pairs (DP-G) from the data.

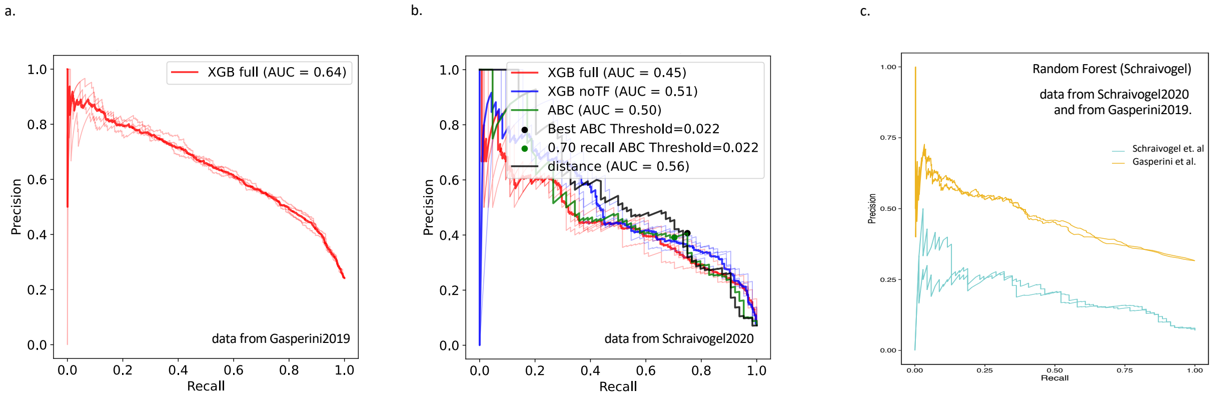

Supplementary Figure S17. Comparison with earlier study Schraivogel2020. Precision-Recall Curves produced based on the algorithms and data used in Schraivogel2020. a) application of XGB of this paper applied to data from Gasperini2019 filtered as described in Schraivogel2020. b) application of XGB applied to data produced through TAPseq from Schraivogel 2020. c) application of the supervised Random Forest algorithm introduced in Schraivogel2020. classifiers trained on the dataset from Schraivogel2020 and applied to the dataset from Gasperini2019 (orange line), or classifiers trained on the dataset from Gasperini2019 and applied to dataset from Schraivogel2020 (blue line).

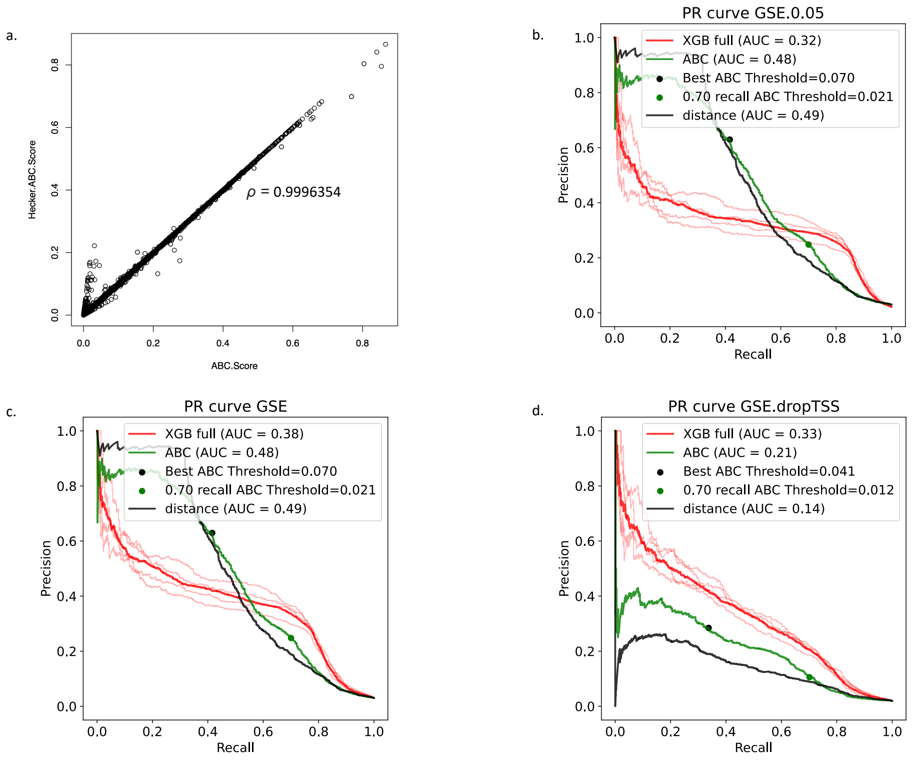

Supplementary Figure S18. Comparison with earlier study Hecker2023. a) comparison of ABC score values in our study to the ABC score values in the study of Hecker et al. 2023 with candidate enhancers defined as in Fulco2019 b) XGB and ABC applied to the EP pairs of Gasperini2019 reported in Hecker2023. This includes the positive controls targeting TSS, and uses adjusted p-value < 0.05 as the threshold for significance. c) XGB and ABC applied to the EP pairs of Gasperini2019 reported in Hecker2023 using significance threshold of adjusted p-value < 0.1 as in Gasperini2019. d) XGB and ABC applied to the EP pairs of Gasperini2019 reported in Hecker2023 using significance threshold of adjusted p-value < 0.1 and excluding the positive controls targeting TSS.

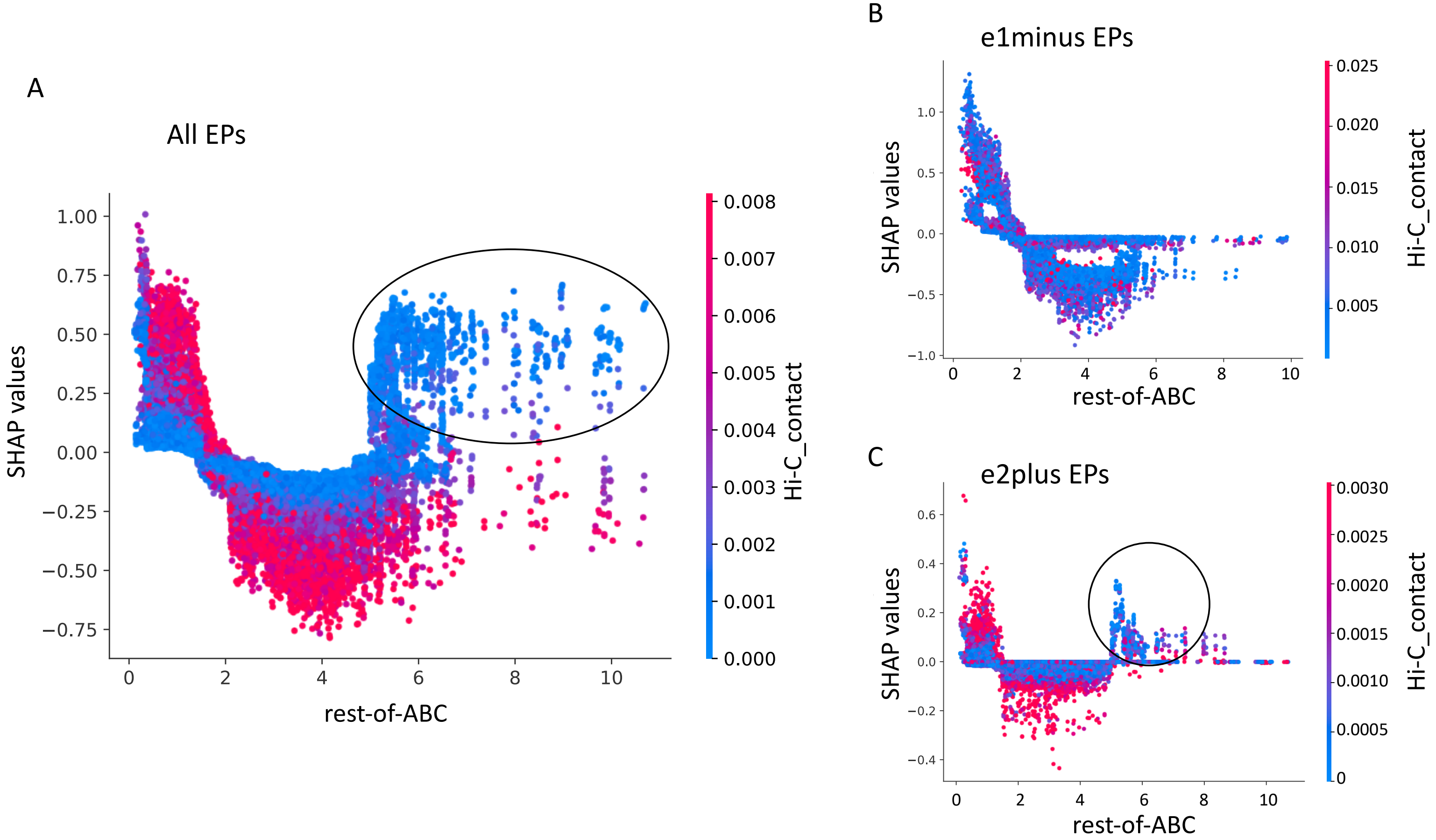

Supplementary Figure S19. **A distinct class of functional EP pairs revealed based on the feature rest-of-ABC. A)** SHAP values for the feature rest-of-ABC (ABC denominator – numerator) of the model trained on the total dataset. For the majority of the data, large value of rest-of-ABC predicts nonfunctional EP pairs, except for this subset of EPs highlighted with an oval. **B)** The model trained on EP pairs with direct contact (e1minus EPs) is missing this pattern. **C)** This pattern is recaptured in the model trained on EP pairs without direct contact (e2plus EPs).

Supplementary Figure 6

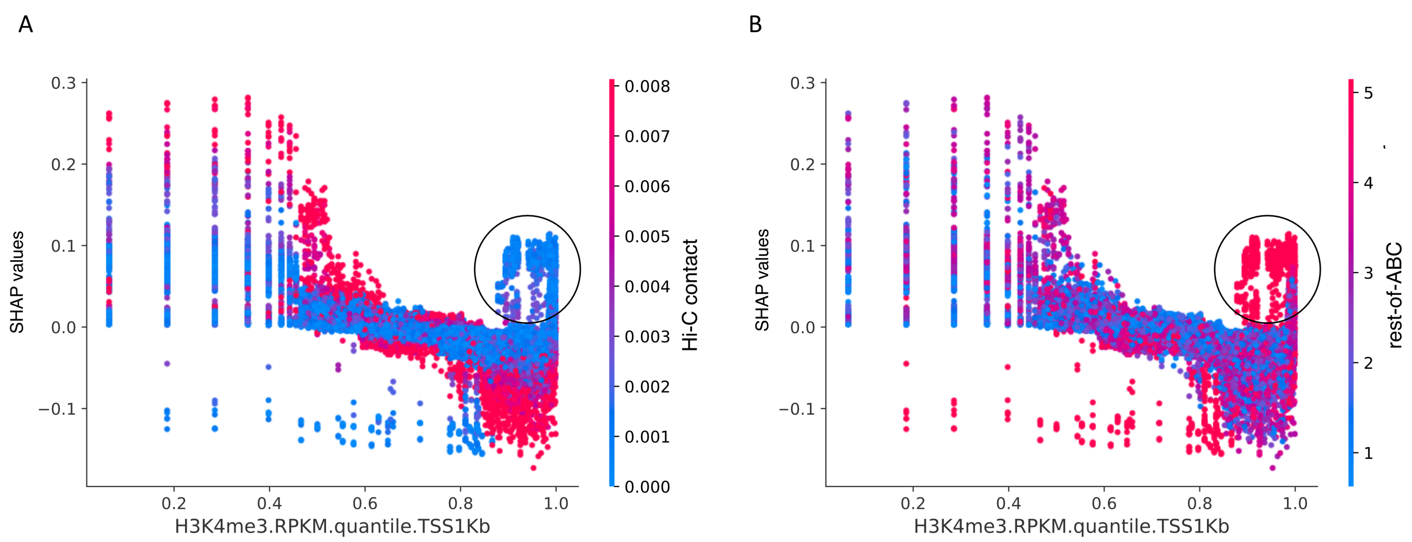

Supplementary Figure S20. **A distinct class of functional EP pairs and H3K4me3 at the promoter.** The same class of indirect but functional EP pairs also show distinct pattern of SHAP values for H3K4me3 at the promoter. **A)** SHAP plot of promoter H3K4me3 marks with color scaled by Hi-C contact between enhancer and promoter and **B)** rest-of-ABC (ABC denominator - numerator). For the rest of the data, strong H3K4me3 at the promoter predicts nonfunctional EP pairs, except for this subset of EPs without direct contact and strong/many enhancers nearby.

References

1. Fulco CP, Nasser J, Jones TR *et al.* Activity-by-Contact model of enhancer specificity from thousands of CRISPR perturbations. *bioRxiv* 2019:529990.

2. Gasperini M, Hill AJ, McFaline-Figueroa JL *et al.* A Genome-wide Framework for Mapping Gene Regulation via Cellular Genetic Screens. *Cell* 2019;**176**:377-390.e19.

3. Rao SSP, Huntley MH, Durand NC *et al.* A 3D Map of the Human Genome at Kilobase Resolution Reveals Principles of Chromatin Looping. *Cell* 2014;**159**:1665–80.

4. Durand NC, Robinson JT, Shamim MS *et al.* Juicebox Provides a Visualization System for Hi-C Contact Maps with Unlimited Zoom. *Cell Syst* 2016;**3**:99–101.

5. Schraivogel D, Gschwind AR, Milbank JH *et al.* Targeted Perturb-seq enables genome-scale genetic screens in single cells. *Nat Methods* 2020;**17**:629–35.

6. Whalen S, Schreiber J, Noble WS *et al.* Navigating the pitfalls of applying machine learning in genomics. *Nat Rev Genet* 2022;**23**:169–81.

7. Xi W, Beer MA. Local epigenomic state cannot discriminate interacting and non-interacting enhancer–promoter pairs with high accuracy. *PLOS Comput Biol* 2018;**14**:e1006625.

8. Chen T, Guestrin C. XGBoost: A Scalable Tree Boosting System. *Proceedings of the 22nd ACM SIGKDD International Conference on Knowledge Discovery and Data Mining*. 2016, 785–94.

9. Kursa MB, Rudnicki WR. Feature Selection with the Boruta Package. *J Stat Softw* 2010;**36**:1–13.

10. Akiba T, Sano S, Yanase T *et al.* Optuna: A next-generation hyperparameter optimization framework. 2019, 2623–31.

11. Kaul A, Bhattacharyya S, Ay F. Identifying statistically significant chromatin contacts from Hi-C data with FitHiC2. *Nat Protoc* 2020;**15**:991–1012.

12. Song W, Sharan R, Ovcharenko I. The first enhancer in an enhancer chain safeguards subsequent enhancer-promoter contacts from a distance. *Genome Biol* 2019;**20**:197.

13. Wysocka J, Myers MP, Laherty CD *et al.* Human Sin3 deacetylase and trithorax-related Set1/Ash2 histone H3-K4 methyltransferase are tethered together selectively by the cell-proliferation factor HCF-1. *Genes Dev* 2003;**17**:896–911.

14. Hughes-Davies L, Huntsman D, Ruas M *et al.* EMSY Links the BRCA2 Pathway to Sporadic Breast and Ovarian Cancer. *Cell* 2003;**115**:523–35.

15. Varier RA, de Santa Pau EC, van der Groep P *et al.* Recruitment of the Mammalian Histone-modifying EMSY Complex to Target Genes Is Regulated by ZNF131 *. *J Biol Chem* 2016;**291**:7313–24.

16. Tyagi S, Chabes AL, Wysocka J *et al.* E2F Activation of S Phase Promoters via Association with HCF-1 and the MLL Family of Histone H3K4 Methyltransferases. *Mol Cell* 2007;**27**:107–19.

17. Bergman DT, Jones TR, Liu V *et al.* Compatibility rules of human enhancer and promoter sequences. *Nature* 2022;**607**:176–84.

18. Hempel M, Cremer K, Ockeloen CW *et al.* De Novo Mutations in CHAMP1 Cause Intellectual Disability with Severe Speech Impairment. *Am J Hum Genet* 2015;**97**:493–500.

19. Itoh G, Kanno S, Uchida KSK *et al.* CAMP (C13orf8, ZNF828) is a novel regulator of kinetochore–microtubule attachment. *EMBO J* 2011;**30**:130–44.

20. Li F, Sarangi P, Iyer DR *et al.* CHAMP1 binds to REV7/FANCV and promotes homologous recombination repair. *Cell Rep* 2022;**40**, DOI: 10.1016/j.celrep.2022.111297.
