# Supplementary figures and images for: Supervised learning of enhancer–promoter specificity based on genome-wide perturbation studies highlights areas for improvement in learning
Source: Bioinformatics. 2024 Jun 13;40(6):btae367. doi: 10.1093/bioinformatics/btae367 (PMC11211214; doi:10.1093/bioinformatics/btae367)

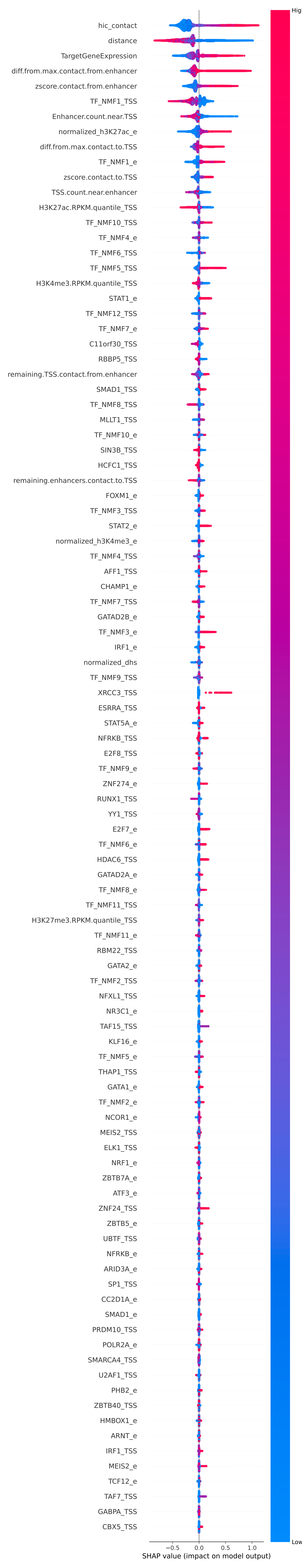

Supplement: btae367_Supplementary_Data [file btae367_supplementary_data.zip › SFigure5.Top100shap.pdf]

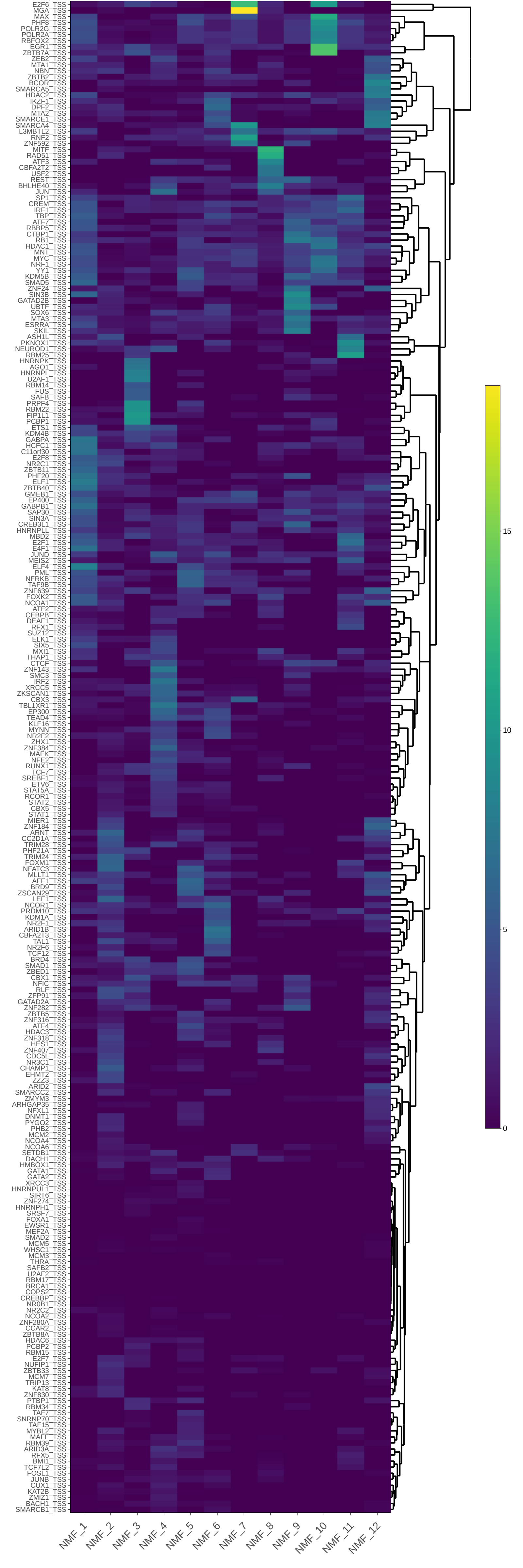

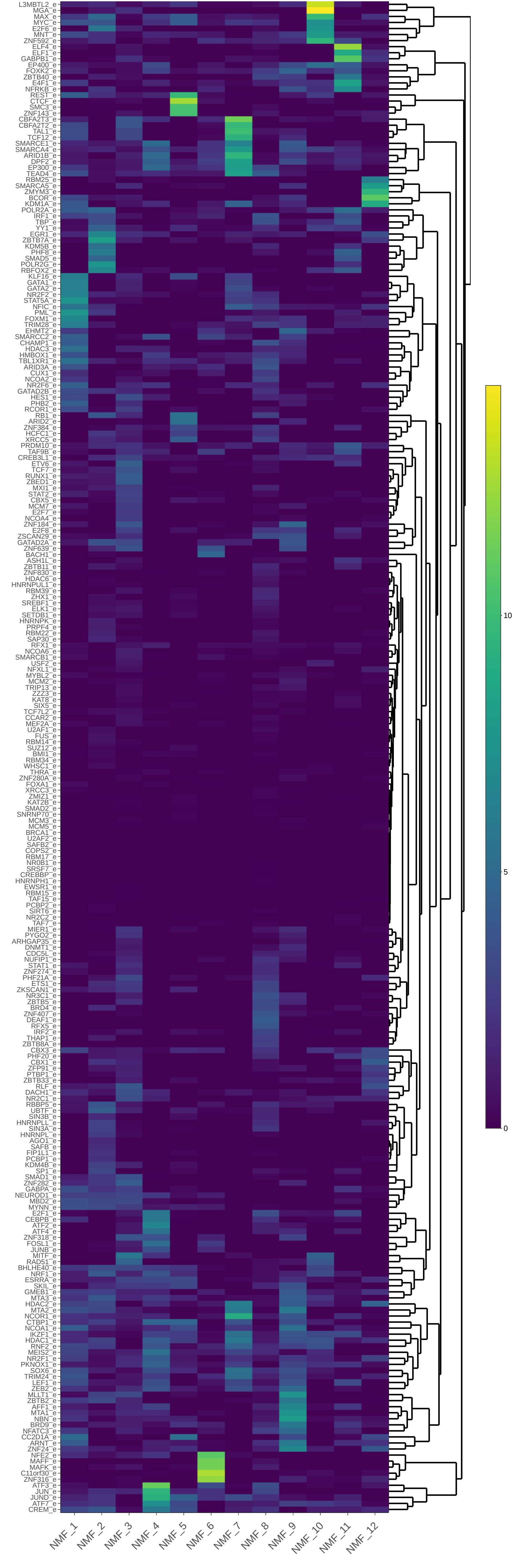

Supplement: btae367_Supplementary_Data [file btae367_supplementary_data.zip › SFigure8.NMF.pdf]
